# Unlocking the Potential of Sea Bass Skin: Characterization of Gelatin and Edible Gelatin Films

**DOI:** 10.1002/fsn3.72333

**Published:** 2026-09-21

**Authors:** Mehmet Gokcin, Yasemen Yanar, Cigdem Dikel

**Affiliations:** ^1^ Department of Seafood Processing Technology Faculty of Fisheries, Cukurova University Adana Turkey; ^2^ Department of Biotechnology Cukurova University Adana Turkey

**Keywords:** by‐products valorization, edible film, gelatin, physicochemical characterization, sea bass skin

## Abstract

This research focused on the extraction of gelatin from the skin of European sea bass ( *Dicentrarchus labrax* ) to engineer edible films, comparing their functional properties against standard bovine and fish‐derived commercial gelatins. Structural evaluation via Scanning Electron Microscopy (SEM) showed a typical gelatinous morphology, defined by a network of disordered, interconnected filaments. From a biochemical perspective, the extracted material was protein‐rich (90.93%), with low levels of moisture (8.11%), ash (0.31%), and lipids (0.13%). Amino acid profiling confirmed a glycine‐heavy composition (27.27%) and an imino acid content of 18.71%, contributing to a measured gel strength of 189.5 g. Furthermore, FTIR spectroscopy validated the presence of primary amide bands (A, B, I, II, and III), mirroring the spectral fingerprints of established commercial standards. The resulting glycerol‐plasticized films demonstrated robust mechanical performance, characterized by a tensile strength of 6.53 MPa and an elongation at break of 94.55% at a thickness of 0.14 mm. These results confirm that sea bass skin serves as an effective biopolymer matrix for creating stable, functional packaging. This study highlights the viability of upcycling marine by‐products as high‐performance, sustainable alternatives to mammalian‐based materials in the production of bio‐packaging.

## Graphical abstract

Sea bass ( *Dicentrarchus labrax* ) skin waste was successfully valorized into high‐purity gelatin. SSG exhibited significantly higher oil binding capacity than commercial bovine and fish gelatins. Edible films developed from sea bass skin displayed a smooth, compact, and ordered matrix morphology. The high water solubility of the films suggests a potent sustainable alternative to mammalian‐based packaging. Physicochemical and structural properties were found to be consistent with international commercial standards.

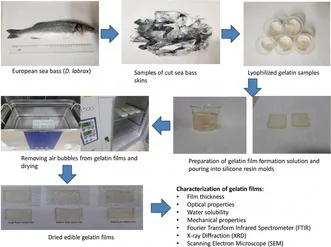

## 1 Introduction

**FIGURE 1:**
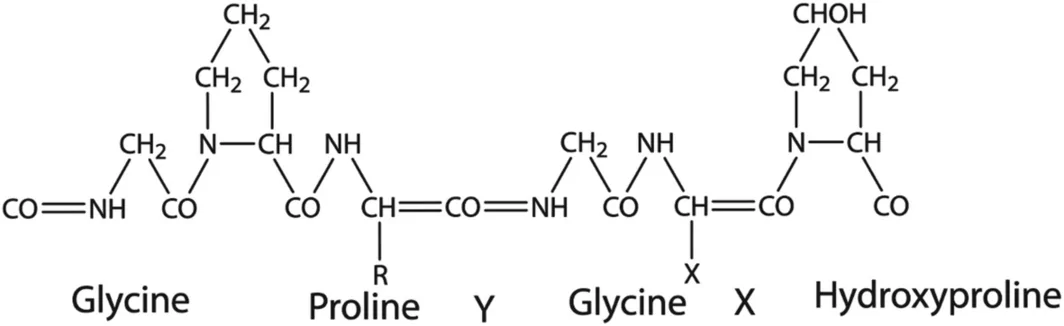
Chemical structure of gelatin.

As a protein‐based polymer, gelatin is susceptible to specific biochemical reactions and can be broken down into constituent amino acids and peptides by various proteolytic enzymes (Mikhailov 2023). Structurally, it retains the characteristic Gly–X–Y repeating motif inherited from collagen, featuring high concentrations of glycine alongside the imino acids proline and hydroxyproline (Figure 1). Furthermore, the molecular mechanisms driving the unique oil‐binding capacity of this specific biopolymer matrix remain fully unmapped. Therefore, this study aims to validate a sustainable valorization pathway for *D. labrax* skin by thoroughly characterizing its physicochemical, structural, and mechanical properties, establishing a definitive baseline for its performance as a competitive, globally inclusive, halal‐compliant bio‐packaging material. Notably, gelatin is frequently employed in reduced‐fat formulations to replicate the creamy mouthfeel and volume typically provided by lipids (Mikhailov 2023). Beyond the pantry, its utility extends into advanced biomedical fields; current research emphasizes its role in tissue engineering, specifically for developing drug delivery vehicles, regenerative medicine frameworks, and scaffolds for cell transition (Zhang et al. 2021; Yang, Yang, et al. 2022). Additionally, gelatin offers advantages, including low immunogenicity, biodegradability, and biocompatibility (Wei et al. 2021). Structurally, gelatin contains an RGD amino acid sequence (Arg–Gly–Asp) that supports cell adhesion (You et al. 2020). At the same time, it contains both single‐ and double‐layered hydrophilic chains, which contribute to its high film‐forming properties (Hanani et al. 2014). Due to the numerous advantageous properties offered by gelatin, it is used in a wide range of applications. Gelatin solutions can form thermoreversible gels on cooling because the chains undergo a conformational disorder–order transition, allowing them to regain the collagen triple helical structure (Bourtoom 2009). Developing edible bio‐based packaging from marine biopolymers offers significant alternatives to replace synthetic petroleum‐based materials (Velhal et al. 2024; Padhan et al. 2026). Gelatin films can be formed by adding plasticizers; gelatin films can be formed and used to encapsulate low‐moisture or fat‐based food components. They can also be used to form films and coatings on meat to protect against oxygen, moisture, and lipid migration (Jorge et al. 2014).

Due to the rapid growth of the global population, demand for gelatin used in food and supplements has increased in recent years, reaching USD 6.51 billion in 2023, and is expected to expand at a compound annual growth rate (CAGR) of 10.1% through 2030 (Grand View Research 2024). Satisfying this escalating industrial demand requires tapping into major regional aquaculture processing centers that generate highly concentrated, traceable streams of clean raw material by‐products.

Skins and ligaments from porcine and bovine sources account for more than 90% of gelatin production on the market (Lin et al. 2017). However, due to certain religious restrictions, gelatin produced from mammalian sources often does not meet halal and kosher food requirements. Additionally, gelatin derived from mammals poses risks due to concerns related to bovine spongiform encephalopathy and foot‐and‐mouth disease (Liao et al. 2021). Consequently, the demand for safer gelatin sources derived from seafood is receiving increasing attention. By‐products of the fish‐processing industry can constitute up to 75% of a fish's total weight and are often used to produce products of low commercial value (such as silage, fertilizers, and fishmeal) or discarded as organic waste in landfills or at sea (Karayannakidis and Zotos 2016). The bioconversion of these by‐products, which typically comprise skin, bones, heads, scales, and viscera, into a functional biopolymer such as gelatin is of paramount importance for modern zero‐waste seafood processing and the circular bioeconomy (Boran et al. 2010; Coppola et al. 2021). The properties of gelatin depend on a variety of factors, such as animal species, age, source tissue, and extraction conditions (Gómez‐Guillén et al. 2011). Gelatin is obtained at higher yields and with better quality from fish skins than from bones (Valcarcel et al. 2021). To date, gelatin extraction and characterization from the skins of various fish species, such as blackmouth catshark ( *Galeus melastomus* ) (Karayannakidis et al. 2024), titan triggerfish ( *B. viridescens* ) (Ahmad et al. 2024), Atlantic cod (Zuev et al. 2023), red skin tilapia (Fazial et al. 2024), grass carp, and sea perch (Ruan et al. 2023), have been reported.

Turkey is a pioneering country in the aquaculture of marine and freshwater products in the Mediterranean region (Demirci et al. 2024). With a production volume of 156,602 tons, European sea bass is the most widely farmed species in aquaculture (Can and Mazlum 2025). Processed sea bass is marketed as fresh, chilled, or filleted in national and international markets. Growing consumer demand for processed seafood products, particularly fish fillets, has led to a proportional rise in processing side‐streams (Siddiqui et al. 2023). After the skinless fillet extraction process, the skins and bones are discarded or used in the feed industry. In marine fisheries and aquaculture sectors, European sea bass represents a high‐value commercial species whose filleting and industrial processing generate substantial volumes of skin side‐streams, accounting for up to 30% of total processing waste (Valcarcel et al. 2021). The strategy is to frame European sea bass processing as a rapidly expanding sector in the Mediterranean basin that creates a massive localized environmental burden. While sea bass gelatin has been extracted before, our study establishes a precise benchmark against both mammalian and commercial cold‐water marine standards using a unique structural and optical cross‐comparison. Furthermore, the molecular mechanisms driving the unique oil‐binding capacity of this specific biopolymer matrix remain fully unmapped. Therefore, this study aims to validate a sustainable valorization pathway for *D. labrax* skin by thoroughly characterizing its physicochemical, structural, and mechanical properties, establishing a definitive baseline for its performance as a competitive, globally inclusive, halal‐compliant bio‐packaging material. Previous studies have primarily focused on the yield or rheological profiling of cultured SSG (Sinthusamran et al. 2015; Valcarcel et al. 2021). Currently, there is a distinct lack of a comprehensive study that directly compares the primary amino acid sequences and imino acid distributions of this species against commercial mammalian and fish skin gelatins, while simultaneously investigating the XRD, SEM, and optical clarity of the resulting gelatin films. The conversion of European sea bass skin into high‐value gelatin—the primary objective of this study—aligns directly with environmental waste management strategies and circular bioeconomy principles. Furthermore, the flexible polypeptide chain conformation facilitates effective penetration and diffusion of the plasticizer during edible film fabrication, enabling the development of cohesive, transparent, and structurally stable edible packaging matrices. In this regard, gelatin, a high‐value natural polymer, was extracted from sea bass skin and comprehensively characterized. Edible films were also prepared from the extracted gelatin.

## 2 Materials and Methods

### 2.1 Materials

Sea bass were purchased from local markets in Adana, Turkey, and brought to the laboratory in insulated boxes filled with ice. The isolated fish skins were collected in a container filled with crushed ice at a skin‐to‐ice ratio of 1:2 (*w*/*w*) to prevent proteolytic degradation. The skins were then washed with cold purified water to remove blood and other residues. Subsequently, they were stored in polyethylene bags at −20°C until the extraction process.

To characterize the extracted gelatin, bovine skin gelatin (CBG; Sigma, CAS No.: 9000‐70‐8), and cold‐water fish skin gelatin (CFG; Sigma, CAS No.: 9000‐70‐8) were used. Commercial bovine skin gelatin (CBG) was selected to serve as the industrial benchmark for high mechanical strength and imino acid density, while cold‐water fish skin gelatin (CFG) was utilized to provide a direct commercial baseline for marine‐sourced alternatives, allowing for an accurate assessment of the structural viability of the extracted SSG matrix. All other chemicals used in this study were of analytical grade and were obtained from Sigma‐Aldrich, Germany.

### 2.2 Sea Bas Skin Gelatin (SSG) Preparation

**FIGURE 2:**
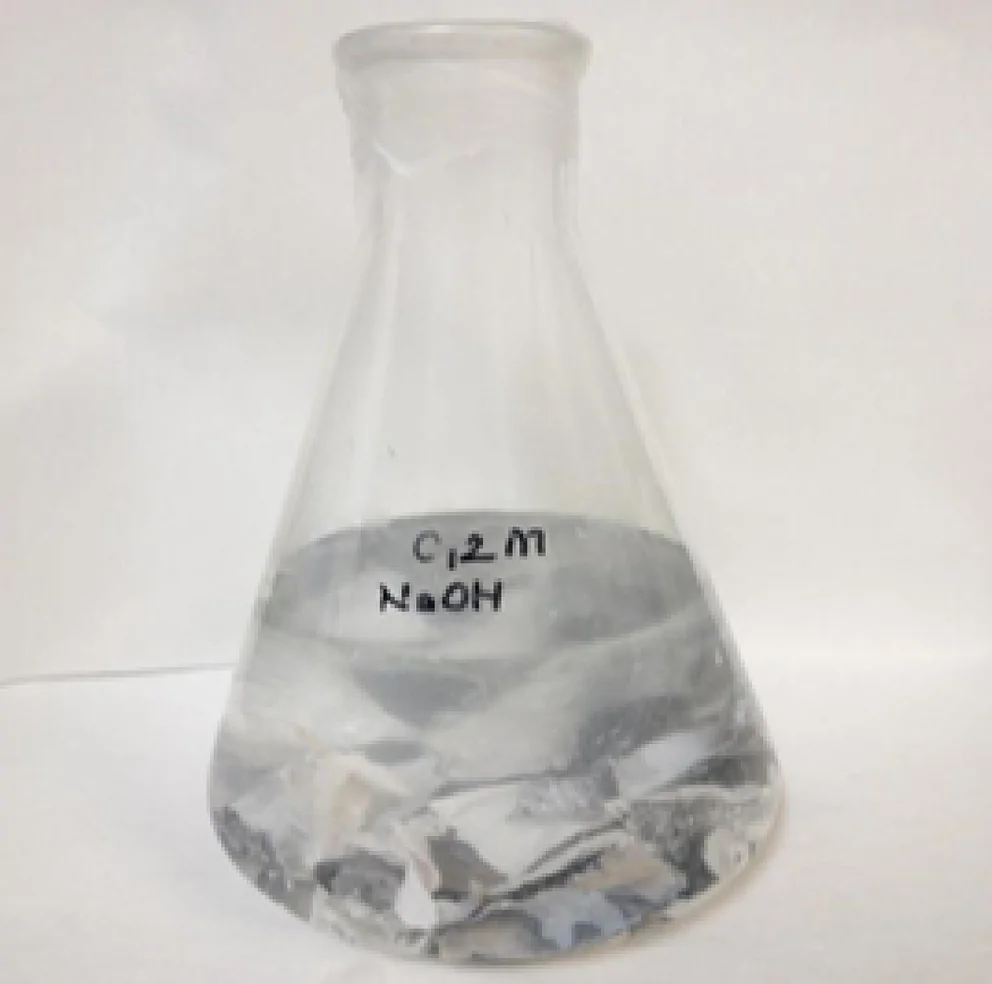
Removal of non‐collagenous proteins from sea bass skin samples.

Sea bass skin samples were washed with purified water and cut into small pieces. The defatting process was repeated multiple times in purified water at 35°C under continuous agitation until no oil particles remained on the surface (Valcarcel et al. 2021). The extremely low final lipid content, coupled with the fact that the reference study performed the water‐agitated defatting process at 22°C, strongly supports that the temperature utilized herein is highly sufficient for removing lipids from fish skins. Furthermore, the applied temperature of 35°C is effective enough to melt and eliminate fish oils while simultaneously preventing the premature leaching and thermal degradation of the native collagen matrix. The efficiency of this chemical‐free step was explicitly validated by the remarkably low remaining lipid content (0.13%). The gelatin extraction was carried out by modifying the method used by Jongjareonrak et al. (2006). To remove non‐collagen proteins and pigments from the skin samples, they were continuously agitated with a 0.2 M NaOH solution at a skin‐to‐solution ratio of 1:10 (Figure 2). Each alkaline treatment was performed for 30 min and then filtered. The remaining water was drained and neutralized by rinsing with tap water until it became neutral to slightly alkaline (pH 7.0–7.5). The skins were then mixed with 0.05 M HCl at a 1:10 skin‐to‐solution ratio and incubated at 25°C for 3 h. The acid‐treated skins were washed with tap water as previously described. The advantage of the sequential dual‐pretreatment application utilized in our study (0.2 M NaOH followed by 0.05 M HCl) lies in its two‐step mechanism: the alkaline treatment effectively eliminates non‐collagenous proteins and pigment impurities, while the subsequent mild acid step disrupts non‐covalent cross‐links, swelling the skin without cleaving the polypeptide backbones. This approach ensures the recovery of a high‐purity polymer rich in alpha chains, which is of paramount importance for achieving stable film formation (Jongjareonrak et al. 2006).

**FIGURE 3:**
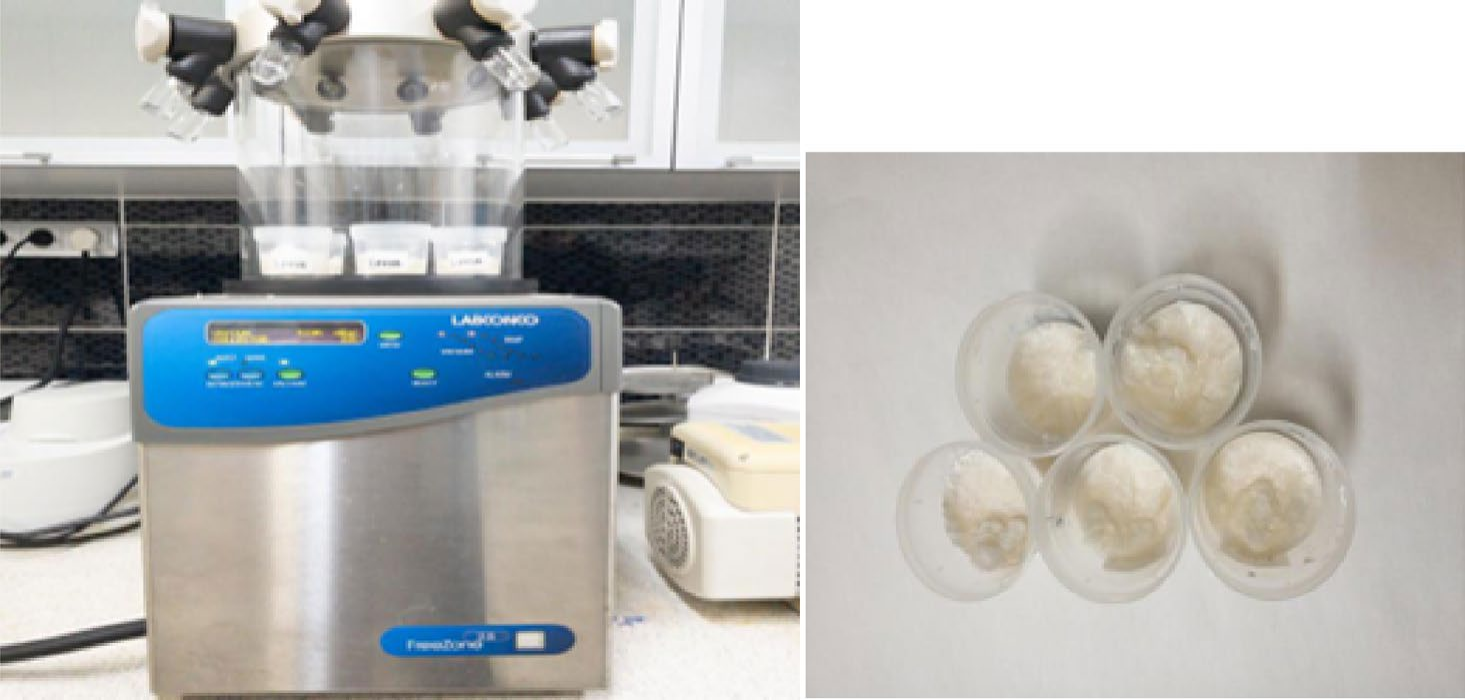
Lyophilization of gelatin samples.

The washed sea bass skins were continuously stirred in distilled water at 45°C, using a skin‐to‐water ratio of 1:10 for 12 h to extract the gelatin. The dissolved gelatin solution was filtered to remove the residual skin fragments. The gelatin was then lyophilized using a lyophilizer (Labconco, Freezone) (Figure 3).

### 2.3 Characterization of Gelatins

#### 2.3.1 Gelatin Yield

The gelatin yield was calculated using the following formula (Jongjareonrak et al. 2006).

$$\text{Gelatin yield}=\frac{\mathrm{Dry}\;\text{gelatin weight}}{\mathrm{Wet}\;\text{fish skin weight}}\times 100$$

#### 2.3.2 Nutritional Composition of Gelatin

The nutritional composition analysis of the gelatin samples was conducted according to the AOAC (2000) method. The crude protein content of the gelatin samples was determined using the Kjeldahl method. To calculate the total protein content, the nitrogen content obtained was multiplied by a nitrogen‐to‐protein conversion factor of 5.4. The moisture content of the gelatin samples was determined by drying the samples at 105°C for 18 h to constant weight and was expressed as a percentage. The ash content was determined by burning the samples at 550°C for 4 h until a constant weight was reached. The lipid content was measured using the Bligh and Dyer method (Bligh and Dyer 1959). All analyses were performed in triplicate.

#### 2.3.3 pH Measurement

The pH values of the gelatin samples were measured using 1% (*w*/*v*, based on protein) gelatin solutions prepared at 60°C, which were allowed to cool to room temperature. Measurements were taken in triplicate using an Orion pH meter, Model 420A (Orion Research Inc., Beverly, MA, USA).

#### 2.3.4 Fourier Transform Infrared Spectrometer (FTIR) Analysis

All spectra of the gelatin powder were obtained using a JASCO ATR Pro One Model 6700 FT/IR spectrometer (JASCO International Co. Ltd., Hachioji, Tokyo, Japan). Measurements were taken over the range 4000–500 cm^−1^, and signals were collected automatically in 32 scans at a resolution of 4 cm^−1^. Spectral data were analyzed using the Spectra Manager TM II cross‐platform software (JASCO).

#### 2.3.5 Determination of Gel Strength

The gel strength of the 6.67% (*w*/*v*) gelatin samples, prepared by dissolving gelatin in distilled water at 60°C and stirring magnetically at 30 rpm (IKA C‐MAG HS 7, Staufen, Germany), was determined according to the AOAC method (AOAC 2000). The samples were placed in bloom jars (Schott, Mainz, Germany), and the gel strength was measured using a standard probe on a TA.XT‐Plus texture analyzer (Stable Micro Systems, Surrey, UK). The maximum force (*g*) was recorded when a penetration distance of 4 mm was reached at a piston speed of 0.5 mm/s.

#### 2.3.6 Melting Point

A 10% (*w*/*v*) gelatin solution was placed into test tubes and stored at 7°C in a refrigerator for 16–18 h before being transferred to a water bath at 10°C. Warm water (45°C) was added gradually, and the temperature at which the gel melted was recorded as the melting point (Muyonga et al. 2004).

#### 2.3.7 Gelling Temperature

To determine the gelling temperature, a 10% gelatin solution was first equilibrated in a 40°C water bath. The system underwent a controlled cooling process, decreasing the temperature at a steady rate of 2°C per minute. Throughout this transition, the thermal profile of the solution was monitored via thermometer at 30‐s intervals. The specific point at which the solution ceased to flow or drip was identified and recorded as the gelling temperature.

#### 2.3.8 Water Holding and Oil Binding Capacity

The water absorption and oil‐binding capacities were assessed following the centrifugal approach outlined by Diniz and Martin (1997). For these measurements, 1 g of the gelatin sample was introduced into centrifuge tubes containing either 50 mL of distilled water or 10 mL of sunflower oil. The resulting mixtures were allowed to equilibrate at room temperature for 1 h. To ensure consistent interaction between the phases, the samples were agitated using a vortex mixer for 5‐s bursts at 15‐min intervals. Following the incubation period, the solutions were processed in a refrigerated centrifuge at 2800× *g* for 20 min to facilitate phase separation for final capacity calculations. The supernatant was separated by filtration through filter paper. The difference between the initial volumes of distilled water/sunflower oil added to the gelatin samples and the volume of the supernatant was measured, and the results were reported as a percentage.

#### 2.3.9 Amino Acid Analysis

The gelatin samples were hydrolyzed under vacuum in 6 N HCl at 110°C for 24 h. Amino acid analysis was performed using a Shimadzu Nexera‐X2 HPLC device. A derivatized 2‐μl sample was injected into the Shimadzu Shim‐pack XR‐ODS II column, which was maintained at 40°C. The mobile phases used were (A) a 1 mM aqueous solution of K_2_HPO_4_ and KH_2_PO_4_, and (B) a 45:40:15 mixture of acetonitrile, methanol, and water. Amino acids were identified and quantified based on their retention times and peak areas relative to the standards.

#### 2.3.10 Scanning Electron Microscope (SEM) Analysis

The surfaces of the gelatin samples were coated with gold–palladium (Au/Pd) to render them conductive at a rate of approximately 2 Å s^−1^. Scanning electron microscopy (SEM) was performed using a Quanta 650 model (FEI, Columbus, Ohio, USA).

### 2.4 Preparation of Gelatin Films

**FIGURE 4:**
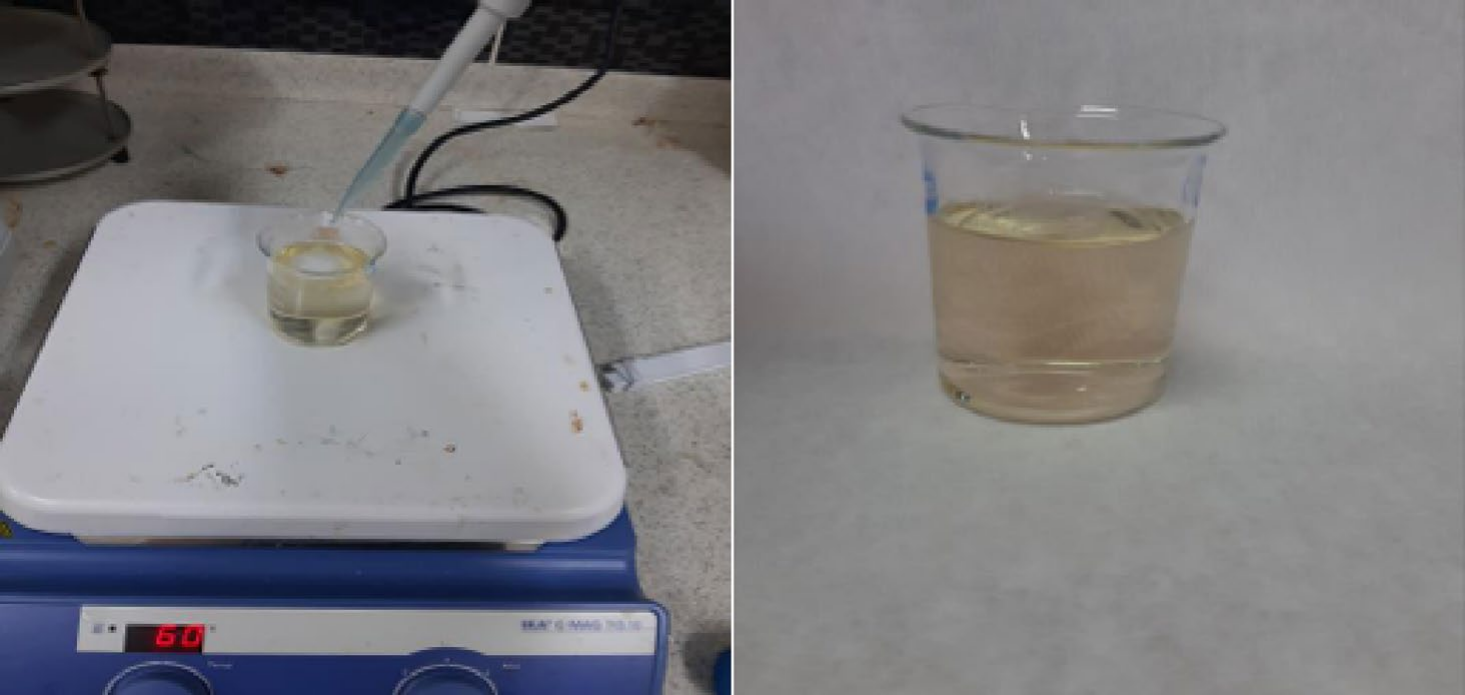
Preparation of gelatin film formation solution.

Gelatin films were prepared according to the method of Jongjareonrak et al. (2006). A gelatin solution was prepared to a 3% protein concentration, and glycerol was added as a plasticizer at 25% relative to the protein concentration. The mixture was then stirred at 60°C for 30 min to create the film‐forming solution (Figure 4). The prepared film‐forming solution was poured into a resin mold (50–55 mm). Air bubbles in the film‐forming solution were removed in an ultrasonic bath (Elmasonic S ultrasonic bath), and the films were dried at 50°C in a Nuve FN500P oven.

#### 2.4.1 Film Thickness

The film thickness was measured with a handheld micrometer Mitutoyo Corp., Model MDC‐25SX, Serial No. 67473329; Kawasaki‐shi, Japan with an accuracy of 0.001 mm. The film was measured at ten random positions. The average of these values was taken as the film thickness.

#### 2.4.2 The Color and Opacity of Films (Optical Properties)

Color measurements of the films were performed using a Hunter Lab Scan (Hunter Associates Laboratory Inc., Reston, VA, USA). The opacity of the films was determined from absorbance readings at 600 nm using a spectrophotometer (Cary 7000, Agilent Technologies, Santa Clara, CA, USA), following the method described by Gómez‐Estaca et al. (2009). Six 1 × 4‐cm film samples were cut and placed in the test cuvette. A blank test cuvette was used as a reference. Opacity was calculated using the following equation:

$$\text{Opacity}\;\left(O\right)=\mathrm{Abs}600/x$$

Abs600 = absorbance value at 600 nm, “*x*” = film thickness (mm).

#### 2.4.3 Water Solubility of Gelatin Films

The water solubility of the films was determined according to the method of Shakila et al. (2012). Films with a surface area of 4 cm^2^ were cut and weighed to determine the initial weight (Ai ±0.0001 g). The films were individually immersed in 15 mL of distilled water and gently agitated at room temperature (22°C–25°C) for 15 h, then filtered through Whatman No. 1 filter paper. The undissolved film fraction collected on the filter paper was dried in a hot‐air oven at 105°C for 24 h and weighed (As). Each film was tested in triplicate, and the mean value was used as the result. The solubility of the film was calculated using the following equation:

$$\text{Solubility}\;\left(\%\right)=\left(\mathrm{Ai}-\mathrm{As}/\mathrm{Ai}\right)\times 100$$

Ai = initial weight of the film, As = undissolved film fraction.

#### 2.4.4 Mechanical Properties

The mechanical properties of the gelatin films, such as tensile strength (TS) and elongation at break (EAB), were determined according to the ASTM (1995) standard method using a TA.XTP plus texture analyzer (Stable Micro Systems Ltd., Surrey, UK). Film thickness was measured using a Mitutoyo digital micrometer (Mitutoyo Corporation, Japan); six measurements were taken to obtain the average thickness. For the test, ten samples (20 × 50 mm^2^) with an initial grip length of 30 mm were pulled at 30 mm/min. At least five replicate measurements were performed for each film, and the mean values were recorded.

#### 2.4.5 Fourier Transform Infrared Spectrometer (FTIR) Analysis

Fourier‐transform infrared (FTIR) spectroscopy of the gelatin films was performed using an FTIR spectrophotometer (JASCO International Co. Ltd., Hachioji, Tokyo, Japan). The films were placed on a crystal cell, and spectra were collected automatically in the range of 4000–500 cm^−1^ with a resolution of 4 cm^−1^ over 32 scans at room temperature. The analysis of the spectral data was performed using the Spectra Manager TM II cross‐platform software (JASCO).

#### 2.4.6 X‐Ray Diffraction (XRD) Analysis

X‐ray diffraction (XRD) analysis was performed to measure the crystalline and amorphous characteristics of the films. An X‐ray diffractometer (Empyrean, Panalytical BV, Almelo, Netherlands) with a copper source was used at room temperature and operated at 40 kV and 30 mA. The diffraction angle range used was 2 θ = 3°–80°; each sample was measured twice.

#### 2.4.7 Scanning Electron Microscope (SEM) Analysis

The surface and cross‐sectional microstructures of the gelatin films were examined using a scanning electron microscope (SEM) Quanta 650 model (FEI (Columbus, Ohio, USA)). Non‐conductive samples were coated with gold–palladium (Au/Pd) at approximately 2 Å s^−1^. The surfaces and cross‐sections of the films were observed at 30 kV.

### 2.5 Statistical Analysis

All collected data were analyzed by analysis of variance (ANOVA), and significant differences among means were determined using Duncan's multiple range test.

## 3 Results and Discussion

### 3.1 Characterization of Gelatin Samples

#### 3.1.1 Gelatin Yield

The yield of gelatin extracted from sea bass skin was 7.88% ± 0.14%. Heating collagen in water at 45°C or higher disrupts its triple‐helix structure, resulting in gelatin, a heterogeneous structure composed of α‐, β‐, and γ‐chains. Therefore, differences in collagen content of the skins of various fish species, as well as variations in gelatin yield reported in the literature, can be attributed to factors such as the types and concentrations of acids used and the extraction methods employed (Sinthusamran et al. 2015; Valcarcel et al. 2021). The gelatin yield in this study is higher than previously reported yields from gray triggerfish ( *Balistes capriscus* ) skin (5.67%) (Jellouli et al. 2011), seabream skin (6.60% ± 0.46%) and seabass skin (6.83% ± 0.39%) (Valcarcel et al. 2021). However, higher yields have also been reported for gelatin extracted from snakehead and shark skins; reported yields ranged from 32.2% ± 1.24% to 33.9% ± 1.60% for snakehead skins and from 31.6% ± 1.02% to 31.1% ± 1.01% for shark skins (Yu et al. 2024).

#### 3.1.2 Nutritional Composition of Gelatin

The nutritional compositions of CBG, CFG, and extracted SSG are shown in Table 1.

**TABLE 1:** Nutrient composition (%) of CFG and CBG compared to gelatin extracted from SSG.

Nutrient components (%)	Gelatins		
	SSG	CFG	CBG
Protein	90.93 ± 0.41^a^	89.31 ± 0.46^b^	90.41 ± 0.42^a^
Moisture	8.11 ± 0.13^b^	9.65 ± 0.18^a^	8.49 ± 0.11^b^
Ash	0.31 ± 0.02^b^	0.20 ± 0.01^c^	0.43 ± 0.04^a^
Lipid	0.13 ± 0.01^b^	0.45 ± 0.02^a^	0.41 ± 0.01^a^
pH	5.66 ± 0.07^a^	5.01 ± 0.04^c^	5.18 ± 0.06^b^

*Note:* ± indicates the standard error. Superscripted letters on numbers in the same row indicate statistically significant differences between groups (*p* < 0.05).

Abbreviations: CBG, commercial bovine skin gelatin; CFG, commercial fish skin gelatin; SSG, sea bass skin gelatin.

The protein content of the extracted SSG (90.93% ± 0.41%) was higher than that of FSG (89.31% ± 0.46%) (*p* < 0.05) and similar to that of BSG (90.41% ± 0.42%) (*p* > 0.05). The high protein content of the gelatin indicates that the extraction was effective. The protein content of the gelatin obtained in our study is higher than that of *Nemipterus japonicus* (72.63%) (Koli et al. 2012). The water content of the extracted SSG (8.11% ± 0.13%) was similar to that of CBG (8.49% ± 0.11%; *p* > 0.05) but lower than that of CFG (9.65% ± 0.18%; *p* < 0.05). Additionally, the fat content of SSG (0.13% ± 0.01%) was lower than that of CFG (0.45% ± 0.02%) and CBG (0.41% ± 0.01%) (*p* < 0.05). Low moisture and fat levels in powdered products increase product stability by reducing microbial growth and oxidative degradation (Kumari et al. 2023). The moisture content of the SSG obtained in this study was lower than the reported value of 10.64% ± 0.45% for *Galeus melastomus* skin gelatin. The fat content was significantly lower than the fat contents reported for gelatin extracted from the skins of four pangasius species ( *Pangasius hypophthalmus* , *Pangasius larnaudii* , *Pangasius larnaudi*, and *Pangasius bocoarti*) (0.77%–1.73%) (Promchote et al. 2018; Karayannakidis et al. 2024). For high‐quality gelatin, the ash content should be below 0.5%. In our study, the ash content of gelatin extracted from SSG (0.31% ± 0.02%) was below this value; it was higher than that of CFG (0.20% ± 0.01%) (*p* < 0.05) but lower than that of CBG (0.43% ± 0.04%) (*p* < 0.05). The ash content of the gelatin obtained in this study was lower than the previously reported value (1.34% ± 0.17%) (Khushboo et al. 2023). These differences in ash content stem from the mineral compositions of the raw materials and extraction methods. Generally, gelatin with an ash content of up to 2.0% is acceptable for food applications (GME 2005).

#### 3.1.3 pH

The pH values of 1% gelatin solutions extracted from SSG, CBG, and CFG are presented in Table 1. Measuring the pH of gelatin is important as it affects other properties such as viscosity and gel strength. In our study, the pH value of the extracted gelatin (pH 5.66) was found to be higher than those of CFG and CBG, exhibiting statistically significant differences across all evaluated groups (*p* < 0.05). These statistical variations in pH are primarily attributable to the chemical pretreatment and washing protocols applied during the extraction process. This value also meets the pH standard of 4.5–6.5 set by the Gelatin Manufacturers Institute of America (GMIA) for edible gelatins (GMIA 2019a, 2019b). Typically, when gelatin is extracted without acid or alkali, a pH value above 6 is expected. The pH of gelatins varies depending on the pre‐treatment conditions applied before extraction (Songchotikunpan et al. 2008). In our study, the method used produced gelatin pretreated with both acid and alkali prior to extraction, resulting in a measured pH of 5.66. The pH value we determined in our study is higher than those reported for gelatins obtained from skins of other fish species using different extraction methods, such as *Channa striatus* (5.39), *Pangasius sutchi* (5.27), *Clarias batrachus* (5.22), and *Oreochromis niloticus* (5.50) (See et al. 2010), *Pangasius pangasius* (5.8), *Hemibagrus nemurus* (5.9), *Oreochromis niloticus* (5.7), and *Channa striata* (5.8) (Ratnasari et al. 2013), and is also higher than reported pH values for *Scoliodon sorrakowah* (4.3), *Labeo rohita* (4.2), and *Katsuwonus pelamis* (4.3) (Shyni et al. 2014). The differences in gelatin pH values also arise from the types and strengths of chemicals used in pre‐treatment.

#### 3.1.4 Fourier Transform Infrared Spectrometer (FTIR) Analysis

**FIGURE 5:**
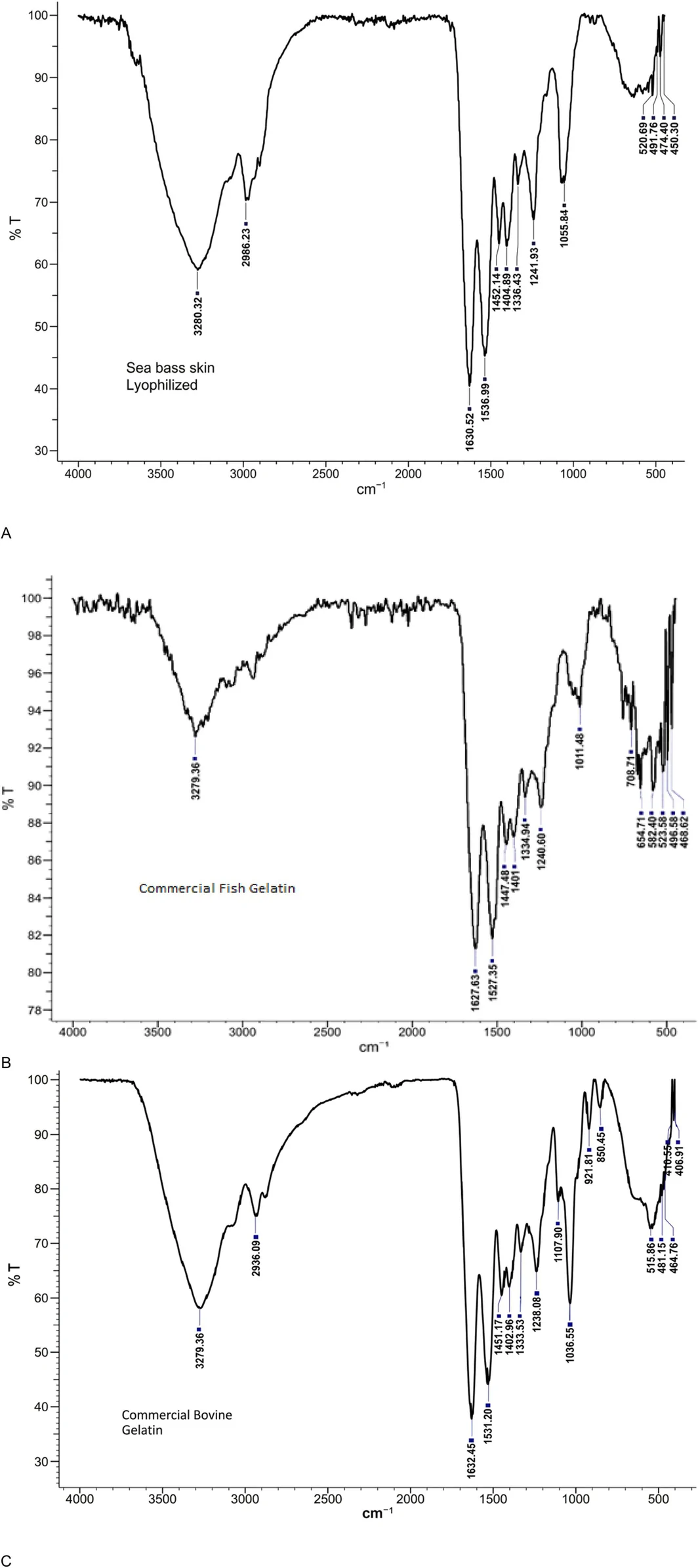
FTIR spectra of SSG (A), CFG (B), and CBG (C).

The FTIR spectrum of the SSG is characterized by several prominent bands corresponding to vibrational transitions in the peptide chains (Azilawati et al. 2015; Derkach et al. 2022). The FTIR analysis was performed on SSG, CBG, and CFG; significant amide bands of the FTIR spectra are shown in Figure 5, and the peak values for the spectral regions and their respective assignments are presented in Table 2. All gelatin samples exhibited similar spectral characteristics, with distinct amide regions. The Amide A band, associated with hydrogen‐bonded N‐H stretching vibrations, was observed at 3280, 3279, and 3279 cm^−1^ in the SSG, CFG, and CBG samples, respectively. Conversely, the slightly higher Amide A frequency observed in SSG confirms the existence of a more flexible, open interaction network, which is highly characteristic of the irregular filamentous arrangement observed via SEM. This spectral behavior perfectly aligns with the broader, more amorphous scattering patterns validated in the 30°–40° (2θ) XRD profiles, chemically corroborating the unique structural conformation and flexibility of these marine biopolymers.

**TABLE 2:** FTIR wavelength peaks and chemical bond relationships in gelatin samples.

Region	SSG	Wave length peaks (cm^−1^)		Relationship
		CFG	CBG	
Amid A	3280.32	3279.36	3279.36	N–H stretching vibration, number of H bonds, and CH_2_ stretching
Amid B	2986.23	2936.09	2971.77	=Asymmetric vibration of CH and –NH_3_ groups
Amid I	1630.52	1627.63	1626.66	C–N stretching C=O stretching vibration, C=O modes interacting with H bonds, CCN deformation and N–H stretching vibration
Amid II	1536.99	1527.35	1517.70	N–H deformation modes and out‐of‐plane stress vibration of C–N groups
Amid III	1241.93	1240.60	1231.33	C–N stretching vibration, N–H deformation modes, and glycine backbone and proline side chain –CH_2_ group vibrations

Kittiphattanabawon et al. (2016) reported that the H bonds present in gelatin are disrupted by high extraction temperatures and/or pretreatments (acid or alkali), leading to the formation of the Amide band at lower frequencies. Similar results have been found for gelatins extracted from black tilapia scales (Sockalingam and Abdullah 2015), *Channa striata* skin and bones (Rosmawati et al. 2021), and catfish skin ( *Pangasius hypophthalmus* ) (Hidayati et al. 2021). In our study, the Amide bands of SSG, CFG, and CBG were found at wavenumbers of 2986.23, 2936.09, and 2987.20 cm^−1^, respectively. The Amide band is associated with the asymmetric stretching vibration of the =C–H and –NH3 groups (Muyonga et al. 2004). The Amide absorption band of SSG is found to be similar to that of CBG, whereas that of CFG is at a lower frequency; this lower frequency has been attributed to the degree of deformation of the collagen secondary structure (Ahmad and Benjakul 2010). The observed amide frequencies for sea bass skin gelatin (SSG) align with established literature for gelatin derived from various marine tissues, including scales and bone (Rosmawati et al. 2021; Nazeer and Kavya Deepthi 2013). Specifically, the Amide I band—a critical indicator of the protein's secondary structure—originated from the stretching vibrations of polypeptide C=O bonds. The intricate contour of this band reflects a superposition of various conformational states within the gelatin chain (Derkach et al. 2022). The Amide I band, which is highly sensitive to carbonyl stretching vibrations along the peptide backbone, shifted to a lower frequency in CBG compared to SSG. This shift indicates a higher density of stable triple‐helix junction zones in CBG, driven by intensive hydrogen bonding that severely restricts backbone flexibility (Yu et al. 2024). Regarding the Amide II region, peaks were identified at 1537 cm^−1^ for SSG, 1527 cm^−1^ for commercial fish gelatin (CFG), and 1518 cm^−1^ for commercial bovine gelatin (CBG). Intensity levels across these samples followed a distinct hierarchy, with SSG showing the highest intensity and CBG the lowest. However, the preservation of the primary structural proteins in SSG is strongly confirmed by the high intensity of the Amide I (1630.52 cm^−1^ and Amide II 1536.99 cm^−1^ bands (Table 2). The Amide I band is highly sensitive to the preservation of carbonyl stretching vibrations within intact peptide backbones. The sharp, unbroadened profile of this region indicates that the low‐temperature hot‐water extraction 45°C for 12 h) effectively prevented thermal over‐hydrolysis into low‐molecular‐weight peptides. This structural integrity is further confirmed by the robust mechanical performance of the prepared films, which exhibited a tensile strength of 6.53 MPa. This, in turn, requires a gelatin matrix inherently dominated by high‐molecular‐weight α‐ and β‐ chain biopolymer components to maintain continuous intermolecular cross‐linking (Muyonga et al. 2004). These values are comparable to the 1539–1586 cm^−1^ range noted by Salem et al. (2023) for *M. mustelus* skin gelatin. Furthermore, the Amide III bands, which are linked to the vibrational modes of the collagen triple‐helix (specifically glycine and proline residues), appeared at 1242 cm^−1^ (SSG), 1241 cm^−1^ (CFG), and 1231 cm^−1^ (CBG). Muyonga et al. (2004) associated the low intensity of the Amide III band with the disruption of the triple‐helical structure during temperature‐induced collagen‐to‐gelatin conversion. Consistent with the FTIR spectra we obtained for all three gelatin samples, Kanwate et al. (2019) reported Amide III bands at 1236, 1239, and 1236 cm^−1^ for freeze‐dried, spray‐dried, and vacuum‐dried fish gelatin samples extracted from the swim bladders of *Labeo rohita* , respectively.

#### 3.1.5 Determination of Gel Strength

Gel strength depends on a variety of factors, such as chemical treatment, extraction temperature, and the size and age of the fish (Shahiri Tabarestani et al. 2014). It is also a function of the ratio of α chains in the gelatin and of the amount of β components, both of which are determined by complex interactions (Gómez‐Guillén et al. 2002). In our study, the gel strength of the gelatin extracted from SSG (189.45 ± 2.10 g) was significantly lower than that of CBG (285.88 ± 0.84 g) and CFG (205.75 ± 3.90 g) (*p* < 0.05) (Table 3). One study reported that the gel strength of gelatin obtained from sea bass skin was lower than that obtained in our study (181.0 ± 7.8 g), whereas gelatin from gilthead sea bream skin and trout skin was reported as 229.5 ± 12.7 g and 95.0 ± 2.0 g, respectively (Valcarcel et al. 2021). Additionally, the gel strength of gelatin derived from Asian sea bass skin has been found to range from 233 to 369 (Sinthusamran et al. 2015). According to the standards of the Gelatin Manufacturers Institute of America (GMIA), the gel strength of commercial gelatin must be between 100 and 300 g for use in the industrial sector (GMIA 2019a). Thus, the gel strength of the gelatin obtained in the present study meets these standards.

**TABLE 3:** Gel strength, water holding and fat binding capacity, melting point, and gelation temperature of gelatin samples.

Parameters	Gelatins		
	SSG	CFG	CBG
Gel strength (g)	189.45 ± 2.10^c^	205.75 ± 3.90^b^	285.88 ± 0.84^a^
Water holding (%)	269.03 ± 5.50^b^	227.44 ± 1.89^c^	312.67 ± 6.76^a^
Fat binding (%)	346.95 ± 5.79^a^	322.59 ± 3.61^b^	287.02 ± 4.41^c^
Melting point (°C)	25.63 ± 0.46^b^	14.02 ± 0.17^c^	32.75 ± 0.51^a^
Gelation temperature (°C)	18.02 ± 0.34^b^	10.93 ± 0.50^c^	23.70 ± 0.23^a^

*Note:* ± indicates the standard error. Letters superscripted on the numbers in the same row indicate statistical differences between groups (*p* < 0.05).

#### 3.1.6 Determination of Melting Point

The melting point of SSG (25.63°C ± 0.46°C) was significantly higher than that of CFG (14.02°C ± 0.17°C) but lower than that of CBG (32.75°C ± 0.51°C) (*p* < 0.05) (Table 3). The gelation temperature of SSG obtained in our study was similar to the gelation temperatures reported for gelatin from triggerfish skin by Ahmad et al. (2024), who used the ultrasonic method (25.96°C and 31.86°C). SSG's lower melting point compared with CBG may be attributable to the lower content of imino acids (proline and hydroxyproline) in sea bass gelatin (Ninan et al. 2014).

#### 3.1.7 Water Holding and Oil Binding Capacity

CBG (312.67%) had the highest water‐holding capacity and the lowest oil‐binding capacity (287.02%) (*p* < 0.05); SSG had the highest oil‐binding capacity (346.95%); and CFG had the lowest water‐holding capacity (227.44%) (*p* < 0.05) (Table 3). The oil‐binding capacity of gelatin is related to its content of hydrophobic amino acids, while its water‐holding capacity is related to its content of hydrophilic amino acids. High water‐holding capacity is primarily associated with increased levels of hydrophilic amino acids and elevated hydroxyproline content (Ninan et al. 2011). Indeed, in our study, CBG with the highest hydroxyproline content also exhibited the highest water‐holding capacity. In this study, the high oil‐binding capacity of SSG is attributed to its tyrosine content; tyrosine is a hydrophobic residue that is more prevalent in SSG than in other gelatins. In the food industry, incorporating gelatin with a high oil‐binding capacity into minced meat products (such as sausages and salami) is desirable not only for fat reduction but also for minimizing drip loss. In this regard, the SSG extracted in our study was more suitable than other gelatin types.

#### 3.1.8 Amino Acid Analysis

The quality and functional characteristics of gelatin are fundamentally dictated by its amino acid distribution. The comparative profiles for SSG, CFG, and CBG are detailed in Table 4. Evaluation of the 17 amino acids analyzed revealed that glycine was the primary constituent across all samples. Specifically, CBG displayed a significantly greater glycine (Gly) concentration (32.68% ± 0.66%) compared to SSG (27.24% ± 1.04%) and CFG (28.95% ± 0.99%) (*p* < 0.05). Proline (Pro) followed as the second most prevalent residue, mirroring the glycine trend with its peak concentration found in CBG (12.88% ± 0.06%; *p* < 0.05). In contrast, no significant difference was observed between the proline levels of SSG (10.55% ± 0.13%) and CFG (10.42% ± 0.24%) (*p* > 0.05), indicating a comparable imino acid profile for the two marine‐derived gelatins. The high concentrations of Gly, Pro, and Ala across all samples are consistent with the amino acid findings reported for silver carp skin and chum salmon skin gelatins (Yang, Tian, et al. 2022; Liu et al. 2023). The amino acid sequence of a gelatin macromolecule can be defined by the general formula Gly–X–Y, where the X position is typically occupied by proline and the Y position by hydroxyproline (Salvatore et al. 2020). Alanine occupies nonpolar regions where Gly–Pro–Y sequences predominate, with the third residue is occupied by either hydroxyproline or alanine. In our study, alanine content was higher in CBG (9.18% ± 0.38%) and CFG (9.49% ± 0.24%) than in SSG (7.43% ± 0.40%) (*p* < 0.05). Generally, mammalian gelatins contain higher levels of imino acids (Pro and Hyp) than fish gelatins (Usman et al. 2025). Supporting this, the imino acid contents of SSG (18.71% ± 0.67%) and CFG (15.20% ± 0.55%) in this study were lower than that of CBG (mammalian) (19.45% ± 0.30%) (*p* < 0.05). The distinct differences in gel strength and melting point observed among the samples are primarily dictated by the primary structure of the polypeptides. As indicated in Table 4, CBG exhibited the highest imino acid content (19.45%), followed by SSG (18.71%) and CFG (15.20%). Structurally, imino acid residues are indispensable for the triple‐helix conformation; the rigid pyrrolidine rings of these imino acids mechanically restrict the conformational flexibility of the polypeptide chains, thereby stabilizing the junction zones during thermoreversible gelation (Joy et al. 2024). Since hydroxyproline acts as a linker for hydrogen bonding through its hydroxyl groups, the significantly higher hydroxyproline content in CBG compared to SSG accounts for the corresponding differences in gel strength (285.88 g vs. 189.45 g) and melting point (32.75°C vs. 25.63°C). Furthermore, although SSG is a fish‐derived gelatin, its imino acid content (18.71%) is statistically closer to that of standard mammalian gelatins than to cold‐water fish gelatin (15.20%), reflecting that the native matrices of *Dicentrarchus labrax* skin gelatin require higher thermal stability.

**TABLE 4:** Amino acid composition of gelatin samples (g/100 g).

Amino acid	Gelatins		
	SSG	CFG	CBG
Glycine	27.24 ± 1.04^b^	28.95 ± 0.99^b^	32.68 ± 0.66^a^
Lysine	3.04 ± 0.14^b^	3.96 ± 0.21^a^	4.04 ± 0.15^a^
Histidine	0.51 ± 0.05^c^	0.63 ± 0.02^a^	0.32 ± 0.01^b^
Arginine	5.41 ± 0.37^b^	6.80 ± 0.32^a^	6.48 ± 0.29^ab^
Aspartic acid	4.21 ± 0.27^b^	5.10 ± 0.19^a^	4.46 ± 0.22^ab^
Glutamic acid	7.39 ± 0.30^b^	9.09 ± 0.24^a^	9.06 ± 0.24^a^
Serine	4.66 ± 0.17^b^	5.40 ± 0.21^a^	3.00 ± 0.01^c^
Tyrosine	0.33 ± 0.05^a^	0.43 ± 0.06^a^	0.09 ± 0.04^b^
Hydroxyproline	6.57 ± 0.24^b^	4.79 ± 0.32^c^	8.16 ± 0.56^a^
Alanine	7.43 ± 0.40^b^	9.49 ± 0.24^a^	9.18 ± 0.38^a^
Valine	0.96 ± 0.03^b^	1.08 ± 0.05^b^	1.78 ± 0.02^a^
Methionine	1.19 ± 0.13^a^	1.36 ± 0.15^a^	1.25 ± 0.01^a^
Phenylalanine	1.40 ± 0.08^b^	1.65 ± 0.06^a^	1.68 ± 0.06^a^
Isoleucine	0.45 ± 0.05^b^	0.62 ± 0.02^a^	0.66 ± 0.02^a^
Leucine	1.57 ± 0.00^c^	2.08 ± 0.03^b^	2.79 ± 0.01^a^
Proline	10.55 ± 0.13^b^	10.42 ± 0.24^b^	12.88 ± 0.06^a^
Tryptophan	0.22 ± 0.01^a^	0.16 ± 0.08^a^	0.17 ± 0.15^a^
Imino acid (Hyp + Pro)	18.71 ± 0.67^a^	15.20 ± 0.55^b^	19.45 ± 0.30^a^
TOTAL	81.34	95.38	97.09

*Note:* ± indicates the standard error. Letters superscripted on the numbers in the same row indicate statistical differences between groups (*p* < 0.05).

The higher tensile strength (TS) of CBG films (8.77 MPa) compared to SSG films (6.53 MPa) is a direct consequence of these dense intermolecular hydrogen bonds (Kozlov and Burdygina 1983). The lower concentrations of glycine (27.24%) and alanine (7.43%) in SSG reduce the frequency of hydrophobic microdomains within the film matrix, leading to a more flexible and less rigid structure.

#### 3.1.9 Scanning Electron Microscope (SEM) Analysis

**FIGURE 6:**
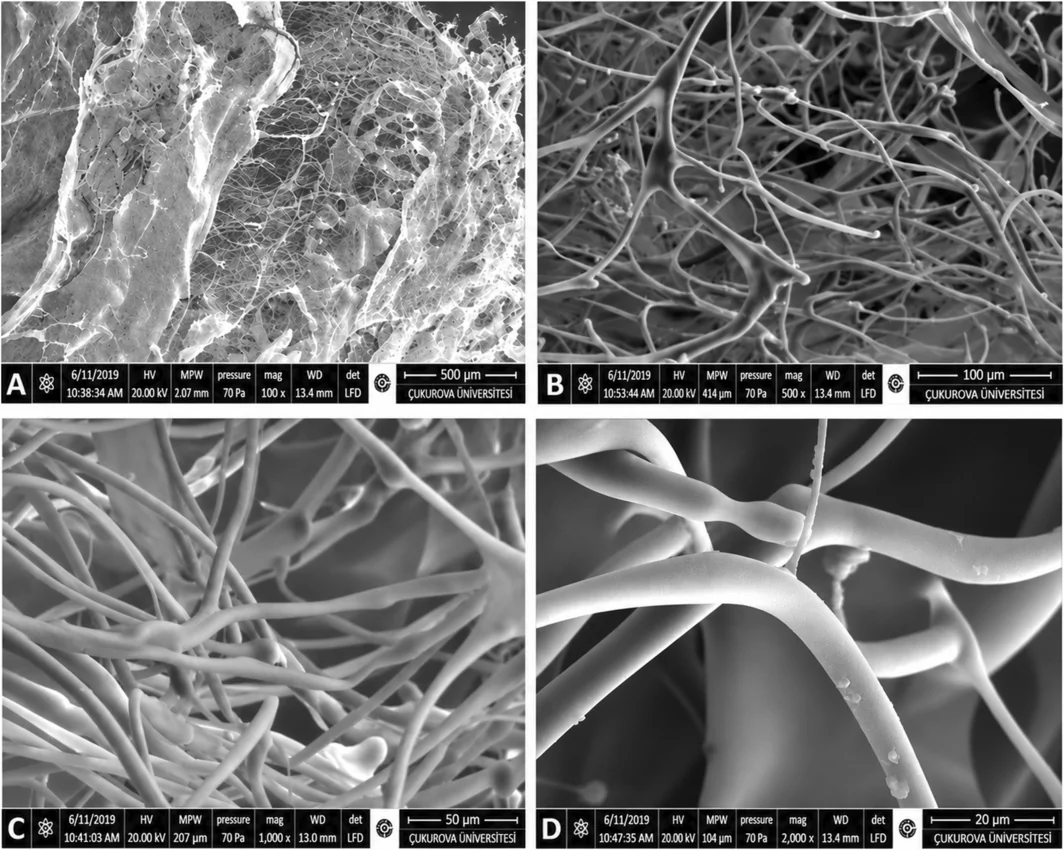
SEM images of lyophilized SSG at ×100 (A), ×500 (B), ×1000 (C), and ×2000 (D) magnification.

**FIGURE 7:**
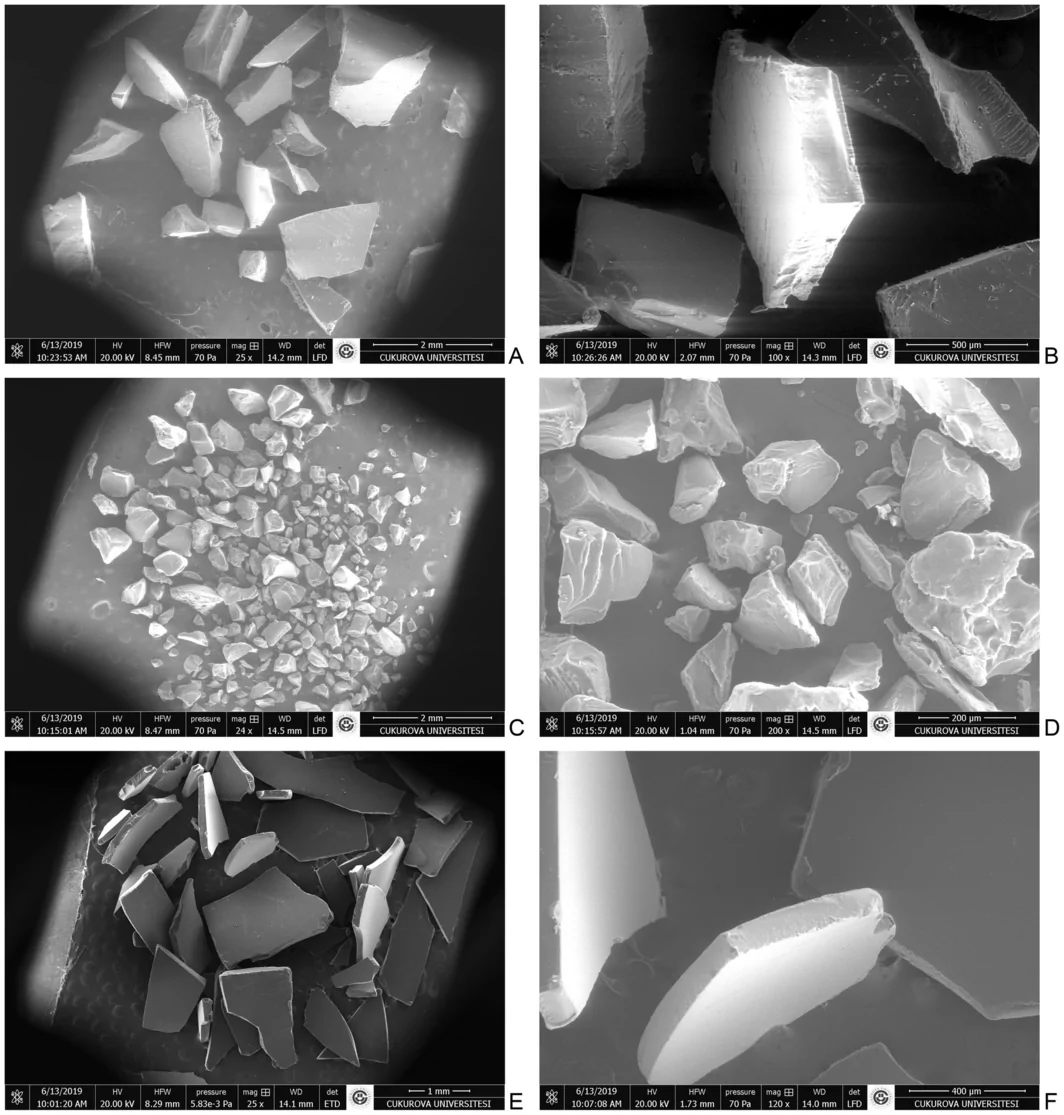
SEM images of gelatin from powdered SSG at ×25 (A), ×100 (B), CBG at ×24 (C), ×200 (D), and CFG at ×25 (E), ×120 (F).

The microstructure of the lyophilized form of SSG was visualized via scanning electron microscopy (SEM) at magnifications of ×100 (A), ×500 (B), ×1000 (C), and ×2000 (D) (Figure 6). Meanwhile, the SEM micrographs of the powdered forms were captured at magnifications of ×25 (A) and ×100 (B) for SSG; ×24 (C) and ×200 (D) for CBG; and ×25 (E) and ×120 (F) for CFG (Figure 7). Upon visual inspection, the lyophilized SSG exhibited a soft, white, sponge‐like appearance with a porous structure. SEM analysis revealed an irregular surface morphology characterized by numerous interconnected, randomly coiled filaments, characteristic of gelatin. This structure is attributed to dehydration during the lyophilization; specifically, lyophilization induces the formation of ice crystals, followed by sublimation under vacuum, resulting in pore formation (Frydrych et al. 2011). Similar observations have been reported for skin gelatins from *Johnius* sp. (Kumar and Rani 2017) and *Brachyplatystoma filamentosum* (Costa da Silva et al. 2017). The inherent porosity of SSG serves as a vital indicator for assessing its potential as a biomaterial, particularly for predicting kinetic profiles in drug release applications. The observed sponge‐like architecture directly impacts the gelatin's functional interactions with liquids; the extensive internal void volume facilitates the high water‐retention capacity characteristic of this material (Usman et al. 2025). When examining the powdered states of all three samples, SEM micro‐graphs revealed irregular particle geometries with surface features resembling fragmented crystalline glass. While the morphological signatures of SSG and CFG powders were largely comparable, CBG was characterized by a reduced particle size and a more rounded, less angular profile. These structural deviations likely stem from the distinct thermal parameters and drying velocities employed during the respective extraction and dehydration phases.

### 3.2 Characterization of Gelatin Films

To fully comprehend the packaging feasibility of the extracted biopolymer, it is of paramount importance to investigate how the baseline biochemical properties of SSG directly dictate the functional and structural parameters of the resulting cast films. The high protein purity (90.93% ± 0.41%) and minimal ash content (0.31% ± 0.02%) of the raw SSG matrix yield a polymer network that facilitates an exceptionally homogenous plasticizer distribution during film preparation. Furthermore, the mechanical and optical properties of these edible films are a direct physical consequence of the primary amino acid distribution described in Table 4. The imino acid content of SSG (18.71% ± 0.67%) functions as the driving force in the formation of the film network, directly dictating the observed film tensile strength (6.53 ± 0.22 MPa) and inducing the smooth, micro‐crack‐free internal structure observed via SEM. Most importantly, the high oil binding capacity validated herein (346.95% ± 5.79%) bridges the gap between a descriptive biochemical study and a practical packaging application. This functional attribute directly accounts for the film opacity levels (0.34 ± 0.02), revealing that SSG is optimized to serve as a specialized packaging material or active coating for high‐fat food systems to protect against lipid migration and oxidation.

#### 3.2.1 Film Thickness

The thicknesses of SSG (0.1403 mm), CFG (0.1388 mm), and CBG (0.1389 mm) films are presented in Table 5 and are statistically similar (*p* > 0.05). Because the primary objective was to compare edible films prepared from extracted SSG with those prepared from CFG and CBG, films were formulated using identical quantities of gelatin and plasticizers. It is generally desirable that the thickness of edible films not exceed 0.25 mm (Shinta et al. 2016); notably, the film thicknesses obtained in this research were well below this threshold. Consistent with our findings, studies on edible films prepared from *Canthidermis maculata* and tilapia ( *Oreochromis niloticus* ) skin and bones reported thicknesses ranging from 0.10 to 0.15 mm. Variations in film thickness are influenced by the gelatin source, the composition of the film solution, and fluctuations in solid content (Zahedi 2019).

**TABLE 5:** Thickness, opacity, L*, a*, and b* color values of gelatin films.

Gelatin films	Color			Opacity	Film thickness (mm)
	L*	a*	b*		
SSG	90.71 ± 0.17^b^	−1.33 ± 0.03^b^	2.84 ± 0.03^b^	0.3398 ± 0.02^a^	0.1403 ± 0.003^a^
CFG	90.76 ± 0.15^b^	−1.30 ± 0.03^b^	2.74 ± 0.08^b^	0.3375 ± 0.02^a^	0.1388 ± 0.05^a^
CBG	95.05 ± 0.45^a^	−0.35 ± 0.02^a^	2.66 ± 0.13^a^	0.2168 ± 0.005^b^	0.1389 ± 0.04^a^

*Note:* ± indicates the standard error. Superscript letters on numbers in the same column indicate statistically significant differences between groups (*p* < 0.05).

#### 3.2.2 The Color and Opacity of Films (Optical Properties)

The colorimetric profiles of the edible films, expressed through Hunter L*, a*, and b* parameters, are detailed in Table 5. Although all samples maintained high transparency, the CBG‐based film demonstrated a significantly superior lightness (L*) value of 95.05 compared to its marine‐derived counterparts (*p* < 0.05). All films displayed negative a* values, suggesting a slight green tint; notably, this green shift was most pronounced in the CBG film at −0.35 (*p* < 0.05). Regarding yellowness (b*), positive values were recorded across all samples, though the CBG film was significantly less yellow (2.66) than the SSG and CFG films (*p* < 0.05). Statistically, the color values (L*, a*, and b*) for the SSG and CFG films were nearly identical, with no significant differences observed (*p* > 0.05). These color variations likely stem from the specific pre‐treatment protocols; while bovine extraction often utilizes 1% HCl (Ahmad et al. 2020), our methodology employed a dual basic and acidic pre‐treatment of the sea bass skin. The parity between our SSG and the commercial fish standard (CFG) validates the efficacy of our extraction technique. Furthermore, the recorded ranges align closely with previous findings for various fish species (Liu et al. 2019; Shahbazi 2017). Finally, opacity measurements indicated that light penetration was highest in the CBG film (*p* < 0.05), whereas SSG and CFG exhibited comparable, higher opacity levels (*p* > 0.05), indicating a slightly more dense internal matrix. Compared to biopolymer composite films prepared by incorporating konjac glucomannan (KGM) into goat skin gelatin matrices, our pure gelatin films exhibited significantly lower opacity values (Hasdar et al. 2024). This optical divergence is directly attributable to fundamental differences in matrix composition and structural cross‐linking behavior. Composite films incorporating polysaccharides such as KGM develop a denser, highly heterogeneous biopolymer network driven by micro‐phase separation and physical intermolecular cross‐linking between proteins and polysaccharides. This structural micro‐heterogeneity markedly enhances light scattering and refraction phenomena across the film polymer matrix. Conversely, the films synthesized in our study formed a more homogeneous and uniform protein gel matrix, thereby imparting superior optical clarity and light transmittance. As coatings for certain food products, edible films are expected to protect against light‐induced degradation. Based on these results, the films prepared from SSG (transparency: 0.34 ± 0.02 A600/mm) and CFG (transparency: 0.34 ± 0.02 A600/mm) are more opaque than the CBG film (transparency: 0.22 ± 0.005 A600/mm) and may therefore be preferable for such applications.

#### 3.2.3 Water Solubility of Gelatin Films

In our study, the edible film prepared with SSG (74.33%) exhibited a solubility similar to that of the CFG film (74.71%) (*p* > 0.05), while both showed significantly higher solubility than the CBG film (72.54%) (*p* < 0.05) (Table 6). Furthermore, the concentration of gelatin in the film‐forming solution and the types of plasticizers (such as glycerol and sorbitol) critically influence the water solubility of the films. In this study, gelatin was incorporated to achieve a 3% protein concentration, and glycerol was used as the plasticizer. Fan et al. (2020) reported solubility values ranging from 89% to 95% for films prepared with protein concentrations between 1% and 7% and a glycerol content equal to 30% of the protein concentration. Consequently, the higher water solubility observed in the edible film prepared from SSG suggests that it may be a suitable alternative to traditional mammalian gelatin films.

**TABLE 6:** Mechanical properties and water solubility of gelatin films.

Gelatin films	TS (MPa)	EAB (%)	Water solubility (%)
SSG	6.53 ± 0.22^b^	94.55 ± 1.64^b^	74.33 ± 0.61^a^
CFG	6.96 ± 0.18^b^	121.89 ± 2.47^a^	74.71 ± 0.73^a^
CBG	8.77 ± 0.13^a^	123.93 ± 2.40^a^	72.54 ± 0.23^b^

*Note:* ± indicates the standard error. Letters superscripted on the numbers in the same column indicate statistical differences between groups (*p* < 0.05).

#### 3.2.4 Mechanical Properties

The mechanical profile of the developed edible packaging films, particularly when evaluated in terms of tensile strength (TS) and elongation at break (EAB), is governed by the density of the triple‐helix cross‐links and the conformation of the gelatin's primary structure. In our study, the TS value of the edible film fabricated with SSG (6.53 ± 0.22 MPa) was statistically comparable to that of the film prepared with CFG (6.96 ± 0.18 MPa; *p* > 0.05), yet significantly lower than that of the CBG‐based film (8.77 ± 0.13 MPa; *p* < 0.05) (Table 6). These values are consistent with the TS values reported for gelatins extracted from various fish species (Bermúdez‐Oria et al. 2017; Jridi et al. 2020). These findings directly reflect the total imino acid content of the source biopolymers; specifically, the higher concentration of proline and hydroxyproline in CBG (19.45%) compared to SSG (18.71%) establishes a denser and more rigid intermolecular hydrogen‐bonding network that robustly resists mechanical deformation under uniaxial tensile stress (Mikhailov 2023; Joy et al. 2024). Conversely, the lower concentration of tightly packed hydrophobic micro‐domains within the SSG matrix stems from its lower glycine (27.24%) and alanine (7.43%) profiles, corresponding to a less rigid and more flexible film structure. Although the lowest EAB value in our study was observed in the SSG film (94.55%), it remains higher than values reported for gelatin films derived from several other fish species (Jridi et al. 2020; Nilsuwan et al. 2021; Said and Sarbon 2022). In their investigation, Shao et al. (2025) reported an elongation at break (EAB) value of 84.06% for control bacterial cellulose‐reinforced konjac glucomannan/gelatin composite films enriched with rosemary and ginger essential oils. This value is notably lower than the EAB recorded for our sea bass skin gelatin (SSG) films (94.55%).

The data indicate that the SSG film exhibited the lowest elongation at break (EAB) value (*p* < 0.05), while the CFG and CBG films showed similar EAB values (*p* > 0.05).

#### 3.2.5 Fourier Transform Infrared Spectrometer (FTIR) Analysis

**FIGURE 8:**
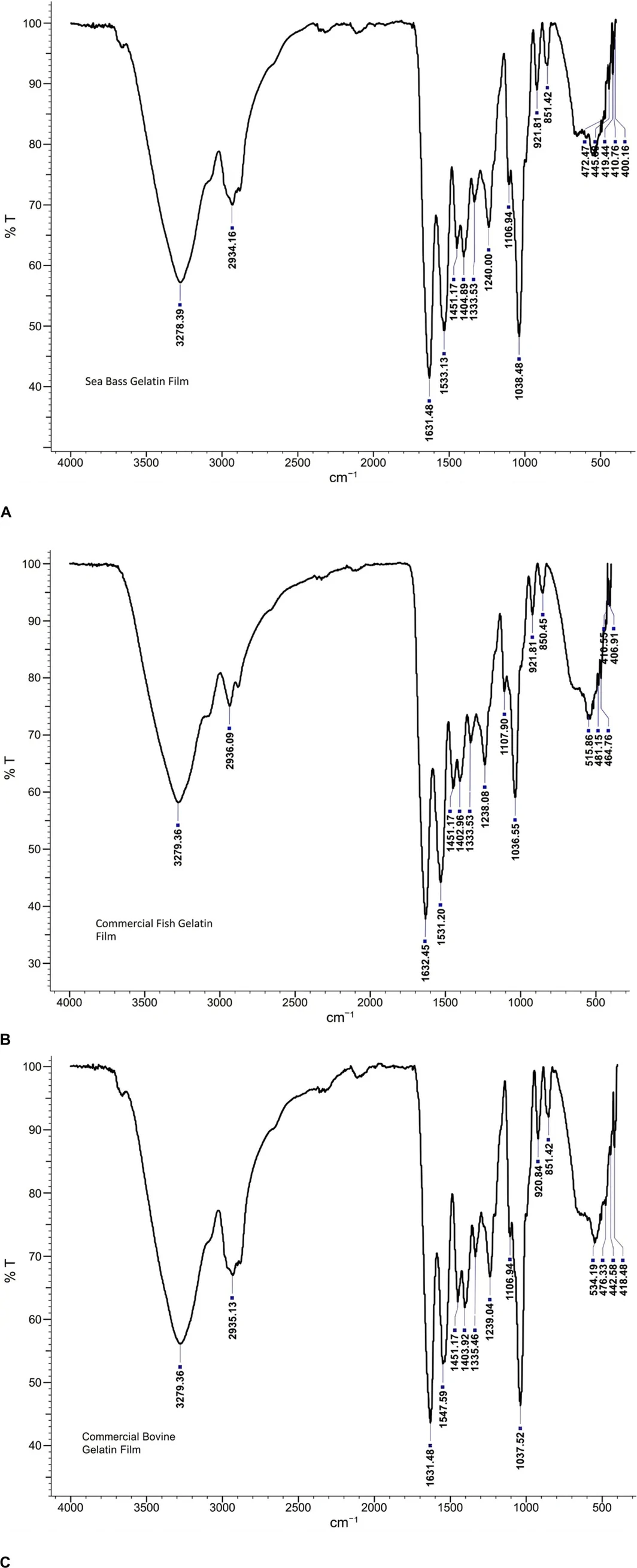
FTIR spectra of films from SSG (A), CFG (B), and CBG (C).

The FTIR spectra of edible films prepared from SSG (A), CFG (B), and CBG (C) gelatins are presented in Figure 8. In general, the spectra of all gelatin films exhibited similar patterns, indicating no significant differences in the functional groups among the gelatin samples. The primary absorption bands in the FTIR spectra of all film samples occur at 1631–1632 cm^−1^ (Amide I: C=O stretching/H‐bonds coupled with COO^−^), 1531–1547 cm^−1^ (Amide II: in‐plane N–H deformation modes and out‐of‐phase C–N stretching vibration), and 1238–1240 cm^−1^ (Amide III: C–N stretching vibration, N–H deformation modes, and –CH_2_ vibrations from the glycine backbone and proline side chains) (Muyonga et al. 2004; Song et al. 2023). In our study, peaks at 850–851, 920–921, 1036–1038, and 1106–1107 cm^−1^ were observed across all three films; these peaks indicate the presence of aliphatic hydroxyl groups originating from glycerol, used as a plasticizer in the gelatin films. Because a standardized amount of glycerol was used, the values obtained for edible films prepared from all three gelatins were closely aligned. Similarly, Basu et al. (2011) reported that the main absorption bands of glycerol, used as a plasticizer in gelatin films, correspond to five peaks: vibrations of C–C bonds at 850, 940, and 1000 cm^−1^, and vibrations of C–O bonds at 1050 and 1100 cm^−1^. Additionally, the Amide A peak at 3278–3279 cm^−1^ corresponds to N–H stretching coupled to hydrogen bonding. The Amide‐B peak, representing the asymmetric stretching vibration of =CH and NH_3_^+^ groups at 2934–2936 cm^−1^, was also observed in the spectra.

#### 3.2.6 X‐Ray Diffraction (XRD) Analysis

Distinct variations were observed across the XRD profiles within the 30°–40° (2θ) bidirectional range. The broad halo at 2θ ≈20° reflects the diffuse scattering of the amorphous matrix; the higher‐angle diffraction features (30°–40°) represent inter‐residue atomic spacing along the single‐stranded polypeptide backbones.

The variations in peak intensity and breadth in this high‐angle region directly correlate with the primary amino acid profiles of the gelatins. Due to its high imino acid density (19.45%), CBG forces tighter, highly repetitive atomic packing along its uncoiled strands. Conversely, the more open, flexible single‐chain conformation of the marine gelatins—particularly the unique, intermediate imino acid arrangement of SSG (18.71%)—creates a slightly more disordered, heterogeneous inter‐residue spacing. This alters the constructive interference of the X‐rays in the 30°–40° range, manifesting as a broader, more attenuated signal that confirms a higher baseline of conformational randomness in the uncoiled marine matrices (Zuev et al. 2023).

**FIGURE 9:**
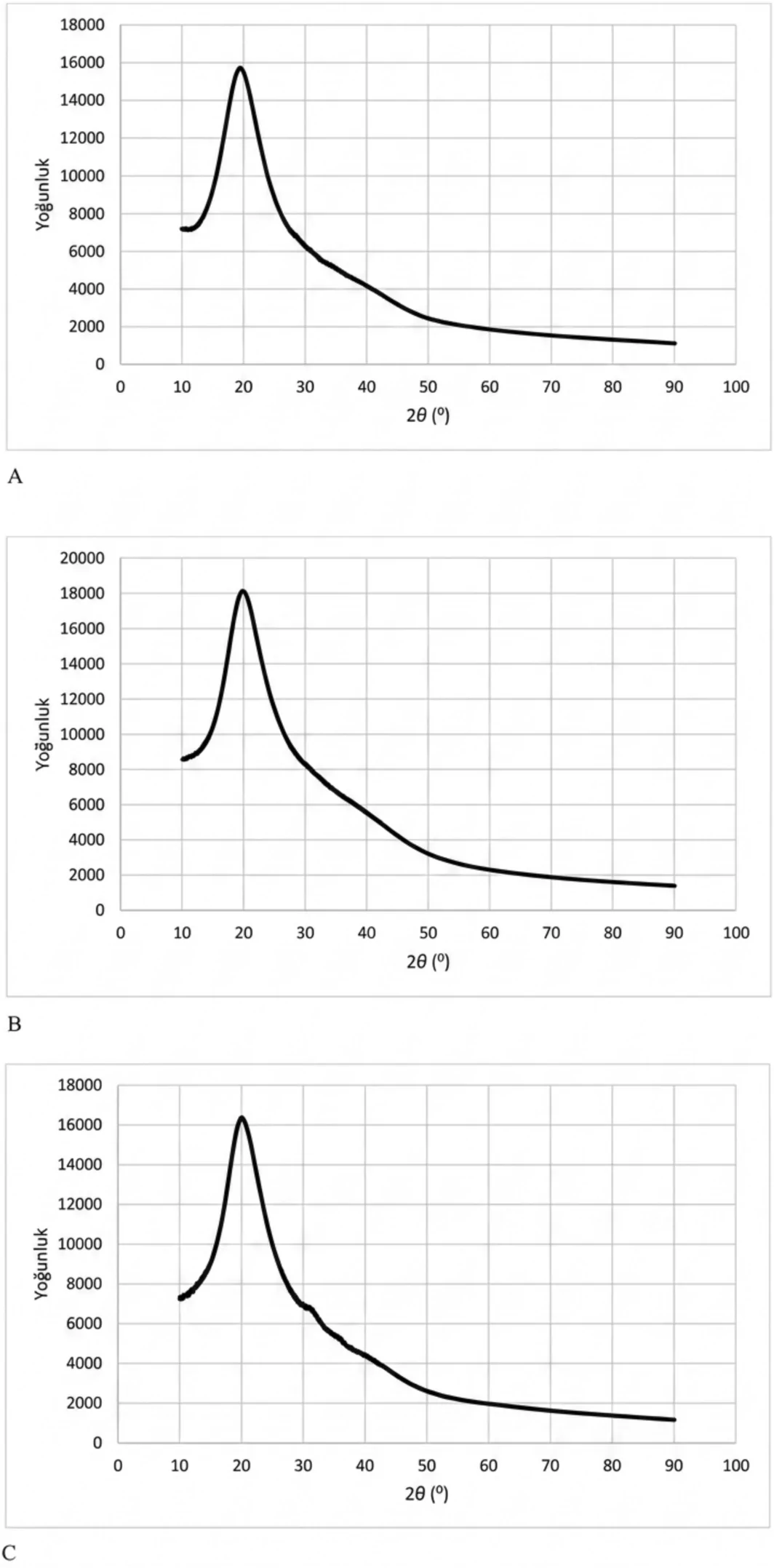
XRD patterns of SSG (A), CFG (B), and CBG (C) films.

All films exhibited an amorphous structure, indicating no tendency toward recrystallization; this is likely due to both the high stability and high moisture content of the gelatin films (Figure 9). The diffractograms of SSG, CFG, and CBG films showed a diffraction peak at approximately 2θ = 20°. The results of this study are consistent with the findings of Gumus et al. (2024), who reported that gelatin films exhibit a broad peak at 2θ = 20°. The addition of glycerol rendered the films more amorphous by reducing the peak intensities at 2θ = 20°. This effect is likely attributable to the high stability of these films following incorporation of glycerol. These results are consistent with Nor et al. (2017), who found that the diffractograms of fish‐skin gelatin films plasticized with glycerol displayed an amorphous pattern.

#### 3.2.7 Scanning Electron Microscope (SEM) Analysis

**FIGURE 10:**
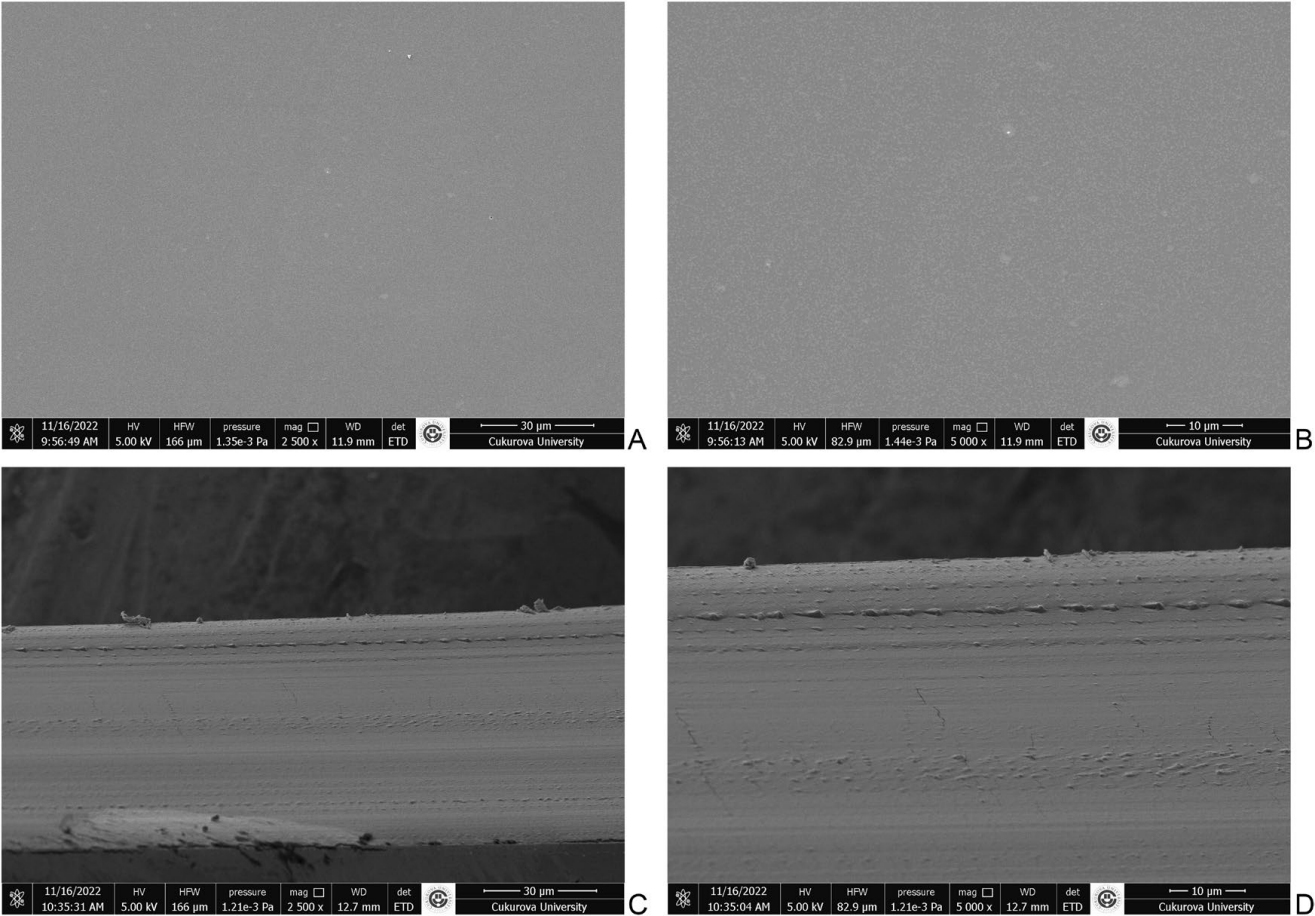
Surface cross‐section ×2500 (A) ×5000 (B) and transverse cross‐section ×2500 (C) ×5000 (D) SEM images of sea bass skin gelatin film.

**FIGURE 11:**
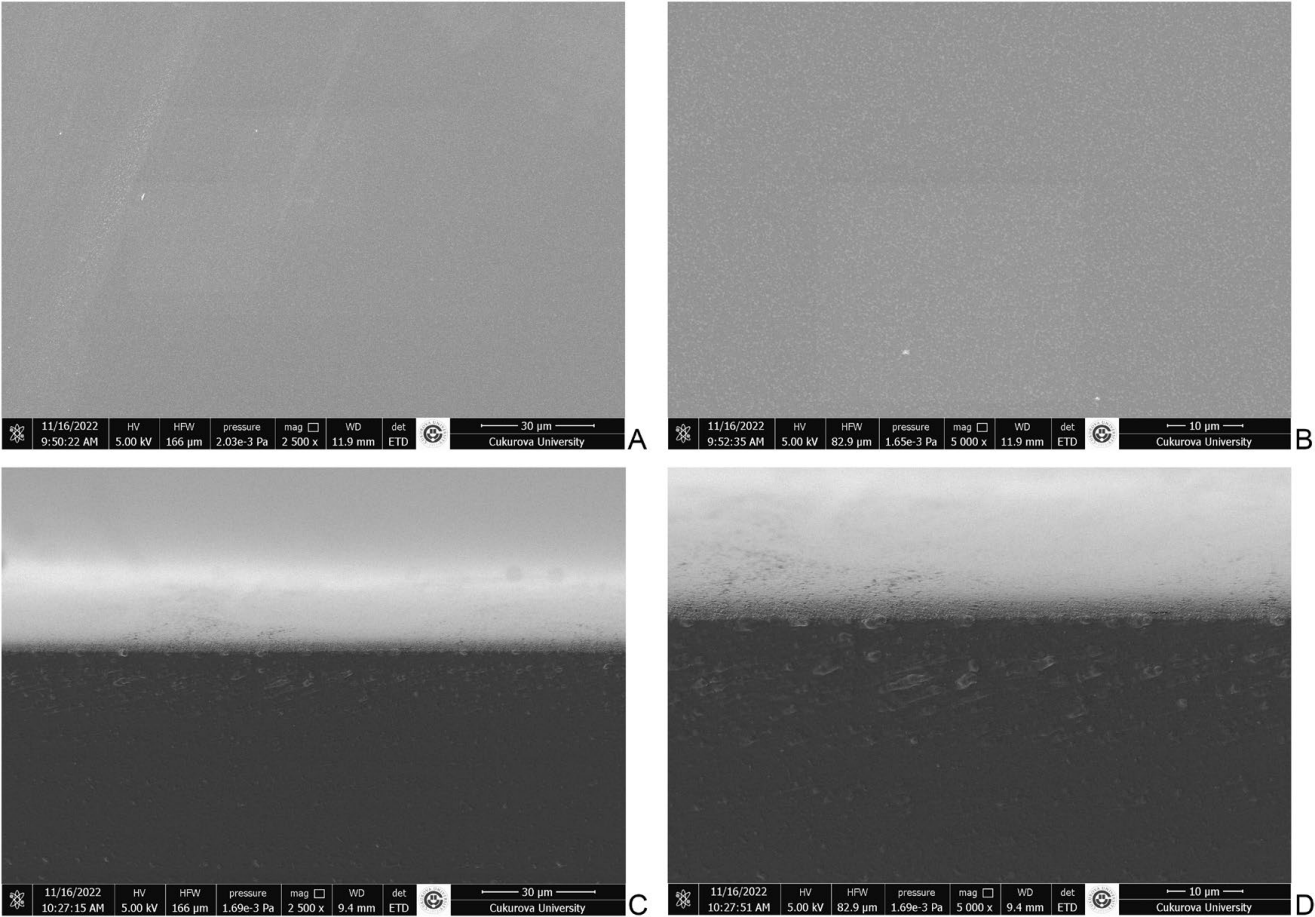
Cross‐section and transverse section of CFG film at ×2500 (A) and ×5000 (B) magnification SEM images at ×2500 (C) and ×5000 (D) magnification.

The microstructural topography and cross‐sectional networks of the developed biopolymer packaging sheets were examined via Scanning Electron Microscopy (SEM) to evaluate matrix homogeneity, phase transitions, and plasticizer integration (Figures 10 and 11). The resulting micrographs revealed notable morphological variations among the film formulations, reflecting an interplay between intrinsic molecular structure and processing conditions.

The film prepared with CBG exhibited a relatively irregular surface characterized by a grainier texture and localized micro‐fissures along its cross‐sectional plane. While mammalian CBG possesses a higher imino acid content (19.45%) that favors the formation of rigid, tightly packed triple‐helical junction zones during casting, this intrinsic structural rigidity is only one contributing factor to the observed micro‐cracks. Beyond molecular composition, processing parameters and environmental dynamics play a vital role during film formation. Beyond intrinsic molecular architecture, film morphology is heavily governed by the interplay of drying kinetics, plasticizer mobility, and environmental conditions during processing. Specifically, rapid or uneven solvent evaporation during drying establishes severe moisture gradients between the surface and core of the film, where rapid capillary shrinkage during water loss generates localized pressure and internal stress that lead to micro‐cracks in dense matrices like CBG. Concurrently, the mobility of glycerol can be restricted within highly aggregated protein domains, resulting in localized plasticizer phase separation or uneven distribution that leaves glycerol‐deficient regions locally brittle and prone to micro‐fissuring under solvent stress.

Conversely, the marine‐derived packaging films—particularly the newly engineered SSG film—outperformed the mammalian standard in structural homogeneity, presenting an exceptionally smooth, uniform, and continuous matrix devoid of macroscopic defects or phase separation (Moreno‐Ricardo et al. 2024). This superior structural integrity is a direct physical consequence of the unique macromolecular architecture of the raw SSG biopolymer. The disordered, randomly coiled filament network characteristic of raw SSG creates a structurally open, accessible polymer network. This spatial configuration allows the small glycerol molecules to thoroughly interpenetrate and establish a highly uniform, continuous network of intermolecular hydrogen bonds across the entire polypeptide matrix.

Furthermore, this structural flexibility moderates drying kinetics by allowing isotropic shrinkage and uniform moisture dissipation during evaporation. Consequently, internal stress build‐up is minimized across both the surface and cross‐sectional planes, preventing localized fracture points. The resulting SSG film displays a cohesive, unified internal topography. This microstructural continuity structurally supports the film's moderate tensile integrity (6.53 MPa) and establishes a dense, channel‐free barrier network capable of minimizing gas and oil diffusion, reinforcing its suitability as a specialized packaging layer for lipid‐retentive food preservation.

### 3.3 Limitations of the Study

Macroscopic properties—including high Bloom strength, functional group preservation in FTIR, and smooth cross‐sectional morphology in SEM—collectively demonstrated high structural integrity. Our future studies involving SDS‐PAGE and gel permeation chromatography (GPC) will focus on quantifying polypeptide chain ratios and further elucidating their precise contributions to mechanical film integrity and cross‐linking mechanisms.

## 4 Conclusions

This research successfully validates the transformation of European sea bass ( *Dicentrarchus labrax* ) skin waste into a high‐value biopolymer for the production of edible gelatin films. The extraction process yielded an SSG with a robust protein content of 90.93%, comparable to the 90.41% found in commercial bovine standards, while maintaining minimal moisture (8.11%), ash (0.31%), and lipid (0.13%) fractions. Spectroscopic analysis via FTIR confirmed that the chemical fingerprint of SSG, particularly within the amide regions, closely aligns with both commercial fish and bovine alternatives. Physicochemical evaluations revealed that while the gel strength of SSG was lower than the commercial benchmarks, its thermal profile—specifically a gelling temperature of 18.02°C—sits within the optimal range for marine gelatins (11°C–28°C). A standout finding of this study was the superior oil‐binding capacity of SSG (346.95%), surpassing both CFG and CBG. This characteristic suggests a high degree of utility in comminuted meat formulations, where high oil retention is essential for fat reduction and the prevention of drip loss. Regarding film performance, all samples maintained a uniform thickness of approximately 0.14 mm, comfortably meeting the industry threshold for edible packaging (< 0.25 mm). The enhanced water solubility of the SSG‐based films further marks them as a viable, fast‐dissolving alternative to traditional mammalian coatings. Ultimately, this study underscores that sea bass processing by‐products, currently viewed as non‐commercial waste, are a high‐potential resource for sustainable bio‐packaging. Transitioning to marine‐sourced gelatin not only mitigates environmental impact but also offers a globally inclusive, halal, and kosher‐friendly alternative to bovine resources.

## Author Contributions

**Mehmet Gokcin:** methodology, investigation, writing – original draft, resources. **Yasemen Yanar:** conceptualization, methodology, supervision, writing – original draft, writing – review and editing, validation, investigation. **Cigdem Dikel:** data curation, writing – original draft, writing – review and editing, investigation, conceptualization, methodology, visualization.

## Funding

This study was supported by the Scientific Research Unit of Cukurova University (Grant FDK‐2017‐9811).

## Conflicts of Interest

The authors declare no conflicts of interest.

## Data Availability

Data are made available upon reasonable request by contacting the corresponding author.

## References

[fsn372333-bib-0001] Ahmad, M. , and S. Benjakul . 2010. “Extraction and Characterisation of Pepsin‐Solubilised Collagen From the Skin of Unicorn Leatherjacket (*Aluterus monocerous*).” Food Chemistry 120, no. 3: 817–824.

[fsn372333-bib-0002] Ahmad, M. , R. Bushra , C. Ritzoulis , H. Meigui , Y. Jin , and H. Xiao . 2024. “Molecular Characterisation, Gelation Kinetics and Rheological Enhancement of Ultrasonically Extracted Triggerfish Skin Gelatine.” Journal of Molecular Structure 1296: 136931.

[fsn372333-bib-0003] Ahmad, T. , A. Ismail , S. A. Ahmad , et al. 2020. “Extraction, Characterization and Molecular Structure of Bovine Skin Gelatin Extracted With Plant Enzymes Bromelain and Zingibain.” Journal of Food Science and Technology 57, no. 10: 3772–3781. 10.1007/s13197-020-04409-2 PMC7447718 32903957

[fsn372333-bib-0004] AOAC . 2000. Official Methods of Analysis of the Association of Official Analytical Chemists. Vol. 11. Association of Official Analytical Chemists.

[fsn372333-bib-0005] ASTM . 1995. ASTM Subcommittee D20.10 on Mechanical Properties. Standard Test Method for Tensile Properties of Thin Plastic Sheeting. American Society for Testing and Materials.

[fsn372333-bib-0006] Azilawati, M. I. , D. M. Hashim , B. Jamilah , and I. Amin . 2015. “RP‐HPLC Method Using 6‐Aminoquinolyl‐N‐Hydroxysuccinimidyl Carbamate Incorporated With Normalization Technique in Principal Component Analysis to Differentiate the Bovine, Porcine and Fish Gelatins.” Food Chemistry 172: 368–376. 10.1016/j.foodchem.2014.09.093 25442566

[fsn372333-bib-0007] Basu, S. K. , K. Kavitha , and M. Rupeshkumar . 2011. “Evaluation of Ionotropic Cross‐Linked Chitosan/Gelatin B Microspheres of Tramadol Hydrochloride.” AAPS PharmSciTech 12, no. 1: 28–34. 10.1208/s12249-010-9537-2 PMC3066349 21161459

[fsn372333-bib-0008] Bermúdez‐Oria, A. , G. Rodríguez‐Gutiérrez , B. Vioque , F. Rubio‐Senent , and J. Fernández‐Bolaños . 2017. “Physical and Functional Properties of Pectin‐Fish Gelatin Films Containing the Olive Phenols Hydroxytyrosol and 3, 4‐Dihydroxyphenylglycol.” Carbohydrate Polymers 178: 368–377. 10.1016/j.carbpol.2017.09.042 29050607

[fsn372333-bib-0009] Bligh, E. G. , and W. J. Dyer . 1959. “A Rapid Method of Total Lipid Extraction and Purification.” Canadian Journal of Biochemistry and Physiology 37, no. 8: 911–917. 10.1139/o59-099 13671378

[fsn372333-bib-0010] Boran, G. , S. J. Mulvaney , and J. M. Regenstein . 2010. “Rheological Properties of Gelatin From Silver Carp Skin Compared to Commercially Available Gelatins From Different Sources.” Journal of Food Science 75, no. 8: E565–E571. 10.1111/j.1750-3841.2010.01543.x 21535497

[fsn372333-bib-0011] Bourtoom, T. 2009. “Edible Protein Films: Properties Enhancement.” International Food Research Journal 16, no. 1: 1–9.

[fsn372333-bib-0012] Can, M. F. , and Y. Mazlum . 2025. “Global Warming and Sustainable Aquaculture in Türkiye: The Case of Sea Bass ( *Dicentrarchus labrax* ) and Gilthead Sea Bream ( *Sparus aurata* ).” Aquaculture International 33, no. 4: 249.

[fsn372333-bib-0013] Coppola, D. , C. Lauritano , F. Palma Esposito , G. Riccio , C. Rizzo , and D. de Pascale . 2021. “Fish Waste: From Problem to Valuable Resource.” Marine Drugs 19, no. 2: 116. 10.3390/md19020116 PMC7923225 33669858

[fsn372333-bib-0014] Demirci, A. , M. F. Can , Y. Mazlum , and E. Şimşek . 2024. “Challenges for Revitalizing Seafood Exports in Hatay's of Türkiye: A Comparative Analysis (2008‐2023).” Ege Journal of Fisheries & Aquatic Sciences (EgeJFAS)/Su Ürünleri Dergisi 41, no. 4: 295–300.

[fsn372333-bib-0015] Derkach, S. R. , D. S. Kolotova , Y. A. Kuchina , and N. V. Shumskaya . 2022. “Characterization of Fish Gelatin Obtained From Atlantic Cod Skin Using Enzymatic Treatment.” Polymers 14, no. 4: 751. 10.3390/polym14040751 PMC8879374 35215662

[fsn372333-bib-0016] Diniz, F. M. , and A. M. Martin . 1997. “Effects of the Extent of Enzymatic Hydrolysis on Functional Properties of Shark Protein Hydrolysate.” LWT ‐ Food Science and Technology 30, no. 3: 266–272.

[fsn372333-bib-0017] Fan, H. Y. , M. J. Dumont , and B. K. Simpson . 2020. “Preparation and Physicochemical Characterization of Films Prepared With Salmon Skin Gelatin Extracted by a Trypsin‐Aided Process.” Current Research in Food Science 3: 146–157. 10.1016/j.crfs.2020.04.002 PMC7473380 32914130

[fsn372333-bib-0018] Fazial, F. F. , N. M. Rasidi , and A. A. A. Ahmad . 2024. “Comparative Analysis of Red Skin Tilapia and Bovine Gelatins as Halal Alternatives in Food Industry.” Halalsphere 4, no. 2: 1–7.

[fsn372333-bib-0019] Frydrych, M. , C. Wan , R. Stengler , K. U. O'Kelly , and B. Chen . 2011. “Structure and Mechanical Properties of Gelatin/Sepiolite Nanocomposite Foams.” Journal of Materials Chemistry 21, no. 25: 9103–9111.

[fsn372333-bib-0020] GME . 2005. “Standard Method for the Testing of Edible Gelatin. Gelatin Monograph.”

[fsn372333-bib-0021] GMIA . 2019a. “GMIA Standard Methods for the Testing of Edible Gelatin.”

[fsn372333-bib-0022] GMIA . 2019b. “Gelatin Manufacturers Institute of America.”

[fsn372333-bib-0023] Gómez‐Estaca, J. , P. Montero , F. Fernández‐Martín , and M. C. Gómez‐Guillén . 2009. “Physico‐Chemical and Film‐Forming Properties of Bovine‐Hide and Tuna‐Skin Gelatin: A Comparative Study.” Journal of Food Engineering 90, no. 4: 480–486.

[fsn372333-bib-0024] Gómez‐Guillén, M. C. , B. Giménez , M. A. López‐Caballero , and M. P. Montero . 2011. “Functional and Bioactive Properties of Collagen and Gelatin From Alternative Sources: A Review.” Food Hydrocolloids 25, no. 8: 1813–1827.

[fsn372333-bib-0025] Gómez‐Guillén, M. C. , J. Turnay , M. D. Fernández‐Dıaz , N. Ulmo , M. A. Lizarbe , and P. J. F. H. Montero . 2002. “Structural and Physical Properties of Gelatin Extracted From Different Marine Species: A Comparative Study.” Food Hydrocolloids 16, no. 1: 25–34.

[fsn372333-bib-0026] Grand View Research . 2024. “Gelatin Market Size, Share & Trends Analysis Report by Source (Bovine, Porcine), by Function (Stabilizer, Thickener), by Application (Food & Beverages, Healthcare), by Region, and Segment Forecasts, 2024–2030.”

[fsn372333-bib-0027] Gumus, T. , G. B. Kaynarca , and D. D. A. Kamer . 2024. “Optimization of an Edible Film Formulation by Incorporating Carrageenan and Red Wine Lees Into Fish Gelatin Film Matrix.” International Journal of Biological Macromolecules 258: 128854. 10.1016/j.ijbiomac.2023.128854 38123042

[fsn372333-bib-0028] Hanani, Z. N. , Y. H. Roos , and J. P. Kerry . 2014. “Use and Application of Gelatin as Potential Biodegradable Packaging Materials for Food Products.” International Journal of Biological Macromolecules 71: 94–102. 10.1016/j.ijbiomac.2014.04.027 24769086

[fsn372333-bib-0029] Hasdar, M. , S. Nalinanon , P. Kittiphattanabawon , and C. Sriket . 2024. “Comprehensive Characterization of Gelatin Films From Goat Skin Incorporating Konjac Glucomannan: Physical, Mechanical, and Molecular Properties.” Indonesian Journal of Science and Technology 9, no. 3: 821–846.

[fsn372333-bib-0030] Hidayati, D. , M. U. Alfarisy , F. Kurniawan , et al. 2021. “Gelatin Extraction Optimization From Skin of Sub Adult and Adult *Pangasius hypophthalmus* .” Current Research in Nutrition and Food Science 9, no. 2: 542.

[fsn372333-bib-0031] Jellouli, K. , R. Balti , A. Bougatef , N. Hmidet , A. Barkia , and M. Nasri . 2011. “Chemical Composition and Characteristics of Skin Gelatin From Grey Triggerfish ( *Balistes capriscus* ).” LWT ‐ Food Science and Technology 44, no. 9: 1965–1970.

[fsn372333-bib-0032] Jongjareonrak, A. , S. Benjakul , W. Visessanguan , and M. Tanaka . 2006. “Skin Gelatin From Bigeye Snapper and Brownstripe Red Snapper: Chemical Compositions and Effect of Microbial Transglutaminase on Gel Properties.” Food Hydrocolloids 20, no. 8: 1216–1222.

[fsn372333-bib-0033] Jorge, M. F. C. , C. H. C. Flaker , S. F. Nassar , I. C. F. Moraes , A. M. Q. B. Bittante , and P. J. do Amaral Sobral . 2014. “Viscoelastic and Rheological Properties of Nanocomposite‐Forming Solutions Based on Gelatin and Montmorillonite.” Journal of Food Engineering 120: 81–87.

[fsn372333-bib-0034] Joy, J. M. , A. Padmaprakashan , A. Pradeep , P. T. Paul , R. J. Mannuthy , and S. Mathew . 2024. “A Review on Fish Skin‐Derived Gelatin: Elucidating the Gelatin Peptides—Preparation, Bioactivity, Mechanistic Insights, and Strategies for Stability Improvement.” Food 13, no. 17: 2793. 10.3390/foods13172793 PMC11394984 39272559

[fsn372333-bib-0035] Jridi, M. , O. Abdelhedi , A. Salem , H. Kechaou , M. Nasri , and Y. Menchari . 2020. “Physicochemical, Antioxidant and Antibacterial Properties of Fish Gelatin‐Based Edible Films Enriched With Orange Peel Pectin: Wrapping Application.” Food Hydrocolloids 103: 105688.

[fsn372333-bib-0036] Kanwate, B. W. , R. V. Ballari , and T. G. Kudre . 2019. “Influence of Spray‐Drying, Freeze‐Drying and Vacuum‐Drying on Physicochemical and Functional Properties of Gelatin From *Labeo rohita* Swim Bladder.” International Journal of Biological Macromolecules 121: 135–141. 10.1016/j.ijbiomac.2018.10.015 30290261

[fsn372333-bib-0037] Karayannakidis, P. D. , S. E. Chatziantoniou , and C. M. Lee . 2024. “Utilization of Blackmouth Catshark ( *Galeus melastomus* ) Skins as an Alternative Source of Gelatin: Extraction and Physicochemical Characterization in Comparison to Porcine Skin Gelatin.” Biomass 4, no. 2: 349–362.

[fsn372333-bib-0038] Karayannakidis, P. D. , and A. Zotos . 2016. “Fish Processing By‐Products as a Potential Source of Gelatin: A Review.” Journal of Aquatic Food Product Technology 25, no. 1: 65–92.

[fsn372333-bib-0039] Khushboo , N. Kaushik , K. N. Widell , R. Slizyte , and A. Kumari . 2023. “Optimization of Single‐Step Gelatin Extraction From Pink Perch ( *Nemipterus japonicus* ) Skin and Bone Obtained From Surimi Industry Using a Green Solvent.” Journal of Food Science 88, no. 12: 5044–5062. 10.1111/1750-3841.16809 37876355

[fsn372333-bib-0040] Kittiphattanabawon, P. , S. Benjakul , S. Sinthusamran , and H. Kishimura . 2016. “Gelatin From Clown Featherback Skin: Extraction Conditions.” LWT ‐ Food Science and Technology 66: 186–192.

[fsn372333-bib-0041] Koli, J. M. , S. Basu , B. B. Nayak , S. B. Patange , A. U. Pagarkar , and V. Gudipati . 2012. “Functional Characteristics of Gelatin Extracted From Skin and Bone of Tiger‐Toothed Croaker ( *Otolithes ruber* ) and Pink Perch ( *Nemipterus japonicus* ).” Food and Bioproducts Processing 90, no. 3: 555–562.

[fsn372333-bib-0042] Kozlov, P. V. , and G. I. Burdygina . 1983. “The Structure and Properties of Solid Gelatin and the Principles of Their Modification.” Polymer 24, no. 6: 651–666.

[fsn372333-bib-0043] Kumar, B. , and S. Rani . 2017. “Technical Note on the Isolation and Characterization of Collagen From Fish Waste Material.” Journal of Food Science and Technology 54, no. 1: 276–278. 10.1007/s13197-016-2443-1 PMC5305709 28242926

[fsn372333-bib-0044] Kumari, A. , N. Kaushik , R. Slizyte , and Khushboo . 2023. “Production and Microencapsulation of Protein Hydrolysate of Pink Perch ( *Nemipterus japonicus* ) By‐Products Obtained From Surimi Industry for Its Sustainable Utilization.” Waste and Biomass Valorization 14, no. 1: 209–226.

[fsn372333-bib-0045] Liao, W. , Y. Zhu , Y. Lu , et al. 2021. “Effect of Extraction Variables on the Physical and Functional Properties of Tilapia Gelatin.” LWT 146: 111514.

[fsn372333-bib-0046] Lin, L. , J. M. Regenstein , S. Lv , J. Lu , and S. Jiang . 2017. “An Overview of Gelatin Derived From Aquatic Animals: Properties and Modification.” Trends in Food Science & Technology 68: 102–112.

[fsn372333-bib-0047] Liu, H. F. , X. W. Pan , H. Q. Li , X. N. Zhang , and X. H. Zhao . 2023. “Amino Acid Composition of a Chum Salmon ( *Oncorhynchus keta* ) Skin Gelatin Hydrolysate and Its Antiapoptotic Effects on Etoposide‐Induced Osteoblasts.” Food 12, no. 12: 2419. 10.3390/foods12122419 PMC10297284 37372630

[fsn372333-bib-0048] Liu, J. , H. Yong , Y. Liu , Y. Qin , J. Kan , and J. Liu . 2019. “Preparation and Characterization of Active and Intelligent Films Based on Fish Gelatin and Haskap Berries ( *Lonicera caerulea* L.) Extract.” Food Packaging and Shelf Life 22: 100417.

[fsn372333-bib-0049] Mikhailov, O. V. 2023. “Gelatin as It Is: History and Modernity.” International Journal of Molecular Sciences 24, no. 4: 3583. 10.3390/ijms24043583 PMC9963746 36834993

[fsn372333-bib-0050] Moreno‐Ricardo, M. A. , P. Gómez‐Contreras , Á. D. González‐Delgado , J. Hernández‐Fernández , and R. Ortega‐Toro . 2024. “Development of Films Based on Chitosan, Gelatin and Collagen Extracted From Bocachico Scales ( *Prochilodus magdalenae* ).” Heliyon 10, no. 3: e25194. 10.1016/j.heliyon.2024.e25194 PMC10839984 38317954

[fsn372333-bib-0051] Muyonga, J. H. , C. G. B. Cole , and K. G. Duodu . 2004. “Extraction and Physico‐Chemical Characterisation of Nile Perch ( *Lates niloticus* ) Skin and Bone Gelatin.” Food Hydrocolloids 18, no. 4: 581–592.

[fsn372333-bib-0052] Nazeer, R. A. , and M. Kavya Deepthi . 2013. “Physicochemical and Nanostructural Properties of Gelatin From Uneconomical Marine Cornet Fish ( *Fistularia petimba* ).” Food Science and Biotechnology 22, no. 1: 9–14.

[fsn372333-bib-0053] Nilsuwan, K. , M. Arnold , S. Benjakul , T. Prodpran , and K. de la Caba . 2021. “Properties of Chicken Protein Isolate/Fish Gelatin Blend Film Incorporated With Phenolic Compounds and Its Application as Pouch for Packing Chicken Skin Oil.” Food Packaging and Shelf Life 30: 100761.

[fsn372333-bib-0054] Ninan, G. , J. Jose , and Z. Abubacker . 2011. “Preparation and Characterization of Gelatin Extracted From the Skins of Rohu ( *Labeo rohita* ) and Common Carp ( *Cyprinus carpio* ).” Journal of Food Processing and Preservation 35, no. 2: 143–162.

[fsn372333-bib-0055] Ninan, G. , J. Joseph , and Z. A. Aliyamveettil . 2014. “A Comparative Study on the Physical, Chemical and Functional Properties of Carp Skin and Mammalian Gelatins.” Journal of Food Science and Technology 51, no. 9: 2085–2091. 10.1007/s13197-012-0681-4 PMC4152545 25190867

[fsn372333-bib-0056] Nor, M. H. , N. N. M. Nazmi , and N. M. Sarbon . 2017. “Effects of Plasticizer Concentrations on Functional Properties of Chicken Skin Gelatin Films.” International Food Research Journal 24, no. 5: 1910–1918.

[fsn372333-bib-0057] Padhan, B. , S. Mukherjee , B. Ray , S. Bhattacharjee , R. Rakshit , and J. Das . 2026. “Animal‐Based Marine Polysaccharides in Food Packaging.” In Multifunctional Marine Polysaccharides: Drug Delivery, Biomedicine and Food Technology Applications, 525–547. Springer.

[fsn372333-bib-0058] Promchote, P. , N. Tongtidram , and P. Supap . 2018. “Properties of Gelatins From Some Selected *Pangasius* Skins.” Food and Applied Bioscience Journal 6: 278–291.

[fsn372333-bib-0059] Ratnasari, I. , S. S. Yuwono , H. Nusyam , and S. B. Widjanarko . 2013. “Extraction and Characterization of Gelatin From Different Fresh Water Fishes as Alternative Sources of Gelatin.” International Food Research Journal 20, no. 6: 3085–3091.

[fsn372333-bib-0060] Rosmawati, R. , A. Bakar Tawali , M. Irfan Said , W. Zzaman , R. Kobun , and N. Huda . 2021. “Characteristics of Gelatin From Skin and Bone of Snakehead ( *Channa striata* ) Extracted With Different Temperature and Time.” Potravinarstvo Slovak Journal of Food Sciences 15, no. 1: 648–661.

[fsn372333-bib-0061] Ruan, Q. , W. Chen , M. Lv , et al. 2023. “Influences of Trypsin Pretreatment on the Structures, Composition, and Functional Characteristics of Skin Gelatin of Tilapia, Grass Carp, and Sea Perch.” Marine Drugs 21, no. 8: 423. 10.3390/md21080423 PMC10456007 37623704

[fsn372333-bib-0062] Said, N. S. , and N. M. Sarbon . 2022. “Physical and Mechanical Characteristics of Gelatin‐Based Films as a Potential Food Packaging Material: A Review.” Membranes 12, no. 5: 442. 10.3390/membranes12050442 PMC9148007 35629768

[fsn372333-bib-0063] Salem, A. , O. Abdelhedi , H. Sebii , et al. 2023. “Techno‐Functional Characterization of Gelatin Extracted From the Smooth‐Hound Shark Skins: Impact of Pretreatments and Drying Methods.” Heliyon 9, no. 9: e19620. 10.1016/j.heliyon.2023.e19620 PMC10558885 37809726

[fsn372333-bib-0064] Salvatore, L. , N. Gallo , M. L. Natali , et al. 2020. “Marine Collagen and Its Derivatives: Versatile and Sustainable Bio‐Resources for Healthcare.” Materials Science and Engineering: C 113: 110963. 10.1016/j.msec.2020.110963 32487384

[fsn372333-bib-0065] See, S. F. , P. K. Hong , K. L. Ng , W. M. Wan Aida , and A. S. Babji . 2010. “Physicochemical Properties of Gelatins Extracted From Skins of Different Freshwater Fish Species.” International Food Research Journal 17, no. 3: 809–816.

[fsn372333-bib-0066] Shahbazi, Y. 2017. “The Properties of Chitosan and Gelatin Films Incorporated With Ethanolic Red Grape Seed Extract and *Ziziphora clinopodioides* Essential Oil as Biodegradable Materials for Active Food Packaging.” International Journal of Biological Macromolecules 99: 746–753. 10.1016/j.ijbiomac.2017.03.065 28315767

[fsn372333-bib-0067] Shahiri Tabarestani, H. , Y. Maghsoudlou , A. Motamedzadegan , and A. R. Sadeghi Mahoonak . 2014. “Effect of Pretreatment Conditions on Physicochemical Properties of Rainbow Trout Skin Gelatin.” Journal of Aquatic Food Product Technology 23, no. 1: 14–24.

[fsn372333-bib-0068] Shakila, R. J. , E. Jeevithan , A. Varatharajakumar , G. Jeyasekaran , and D. J. F. C. Sukumar . 2012. “Comparison of the Properties of Multi‐Composite Fish Gelatin Films With That of Mammalian Gelatin Films.” Food Chemistry 135, no. 4: 2260–2267. 10.1016/j.foodchem.2012.07.069 22980800

[fsn372333-bib-0069] Shao, Z. , W. Lan , and J. Xie . 2025. “A Konjac Glucomannan/Gelatin Film Integrated With Rosemary Essential Oil and Ginger Essential Oil‐Loaded Bacterial Cellulose: Preparation, Characterization, and Application in Sea Bass (*Lateolabrax maculatus*) Packaging.” Food Chemistry 478: 143604. 10.1016/j.foodchem.2025.143604 40043437

[fsn372333-bib-0070] Shinta, D. , A. Supriadi , and S. D. Lestari . 2016. “Pemanfaatan Air Cucian Surimi Belut Sawah ( *Monopterus albus* ) Dalam Pembuatan Edible Film.” Jurnal Fishtech 5, no. 1: 85–93.

[fsn372333-bib-0071] Shyni, K. , G. S. Hema , G. Ninan , S. Mathew , C. G. Joshy , and P. T. Lakshmanan . 2014. “Isolation and Characterization of Gelatin From the Skins of Skipjack Tuna ( *Katsuwonus pelamis* ), Dog Shark (*Scoliodon sorrakowah*), and Rohu ( *Labeo rohita* ).” Food Hydrocolloids 39: 68–76.

[fsn372333-bib-0072] Siddiqui, S. A. , H. Schulte , D. Pleissner , et al. 2023. “Transformation of Seafood Side‐Streams and Residuals Into Valuable Products.” Food 12, no. 2: 422. 10.3390/foods12020422 PMC9857928 36673514

[fsn372333-bib-0073] Silva, E. V. C. D. , L. D. F. H. Lourenço , and R. S. Pena . 2017. “Optimization and Characterization of Gelatin From Kumakuma ( *Brachyplatystoma filamentosum* ) Skin.” CyTA Journal of Food 15, no. 3: 361–368.

[fsn372333-bib-0074] Sinthusamran, S. , S. Benjakul , and H. Kishimura . 2015. “Molecular Characteristics and Properties of Gelatin From Skin of Seabass With Different Sizes.” International Journal of Biological Macromolecules 73: 146–153. 10.1016/j.ijbiomac.2014.11.024 25475845

[fsn372333-bib-0075] Sockalingam, K. , and H. Z. Abdullah . 2015. “Extraction of High Value Added Gelatin Biopolymer From Black Tilapia ( *Oreochromis mossambicus* ) Head Bones.” In AIP Conference Proceedings (Vol. 1669, No. 1, p. 020076). AIP Publishing LLC.

[fsn372333-bib-0076] Song, G. , S. Lin , Y. Wu , et al. 2023. “Emulsifier Free Fish Gelatin Based Films With Excellent Antioxidative and Antibacterial Activity: Preparation, Characterization and Application in Coating Preservation of Fish Fillets.” Journal of Food Engineering 343: 111362.

[fsn372333-bib-0077] Songchotikunpan, P. , J. Tattiyakul , and P. Supaphol . 2008. “Extraction and Electrospinning of Gelatin From Fish Skin.” International Journal of Biological Macromolecules 42, no. 3: 247–255. 10.1016/j.ijbiomac.2007.11.005 18207233

[fsn372333-bib-0078] Usman, M. , J. Mac Regenstein , H. U. U. Rahman , et al. 2025. “Physicochemical and Functional Properties of Rainbow Trout ( *Oncorhynchus mykiss* ) Skin Gelatin in Comparison to Commercial Bovine Gelatin.” Food Chemistry Advances 8: 101088.

[fsn372333-bib-0079] Valcarcel, J. , C. Hermida‐Merino , M. M. Piñeiro , D. Hermida‐Merino , and J. A. Vázquez . 2021. “Extraction and Characterization of Gelatin From Skin By‐Products of Seabream, Seabass and Rainbow Trout Reared in Aquaculture.” International Journal of Molecular Sciences 22, no. 22: 12104. 10.3390/ijms222212104 PMC8620335 34829985

[fsn372333-bib-0080] Velhal, M. , M. Dave , E. Sun , S. Holla , and H. Liang . 2024. “Plant and Animal‐Based Bioderived Materials: A Review of Their Antimicrobial Mechanisms and Applications.” Materials Today Sustainability 27: 100885.

[fsn372333-bib-0081] Wei, B. , W. Wang , X. Liu , et al. 2021. “Gelatin Methacrylate Hydrogel Scaffold Carrying Resveratrol‐Loaded Solid Lipid Nanoparticles for Enhancement of Osteogenic Differentiation of BMSCs and Effective Bone Regeneration.” Regenerative Biomaterials 8, no. 5: rbab044. 10.1093/rb/rbab044 PMC8358478 34394955

[fsn372333-bib-0082] Yang, L. , M. Yang , J. Xu , et al. 2022. “Structural and Emulsion Stabilization Comparison of Four Gelatins From Two Freshwater and Two Marine Fish Skins.” Food Chemistry 371: 131129. 10.1016/j.foodchem.2021.131129 34560337

[fsn372333-bib-0083] Yang, N. , X. Tian , Y. Du , et al. 2022. “Comparative Study of Sodium Nitrite Loaded Gelatin Microspheres and Gelatin Gels: Physicochemical and Antibacterial Properties.” Colloids and Surfaces A: Physicochemical and Engineering Aspects 655: 130309.

[fsn372333-bib-0084] You, S. , S. Liu , X. Dong , H. Li , Y. Zhu , and L. Hu . 2020. “Intravaginal Administration of Human Type III Collagen‐Derived Biomaterial With High Cell‐Adhesion Activity to Treat Vaginal Atrophy in Rats.” ACS Biomaterials Science & Engineering 6, no. 4: 1977–1988. 10.1021/acsbiomaterials.9b01649 33455320

[fsn372333-bib-0085] Yu, E. , C. Pan , W. Chen , et al. 2024. “Gelatin From Specific Freshwater and Saltwater Fish Extracted Using Six Different Methods: Component Interactions, Structural Characteristics, and Functional Properties.” LWT 191: 115656.

[fsn372333-bib-0086] Zahedi, Y. 2019. “Edible/Biodegradable Films and Coatings From Natural Hydrocolloids.” In Emerging Natural Hydrocolloids: Rheology and Functions, 571–599. Wiley.

[fsn372333-bib-0087] Zhang, F. , C. Hu , L. Yang , et al. 2021. “A Conformally Adapted All‐In‐One Hydrogel Coating: Towards Robust Hemocompatibility and Bactericidal Activity.” Journal of Materials Chemistry B 9, no. 11: 2697–2708. 10.1039/d1tb00021g 33683274

[fsn372333-bib-0088] Zuev, Y. F. , S. R. Derkach , L. R. Bogdanova , et al. 2023. “Underused Marine Resources: Sudden Properties of Cod Skin Gelatin Gel.” Gels 9, no. 12: 990. 10.3390/gels9120990 PMC10742947 38131976

